# Impaired immune response mediated by prostaglandin E2 promotes severe COVID-19 disease

**DOI:** 10.1371/journal.pone.0255335

**Published:** 2021-08-04

**Authors:** Melanie Ricke-Hoch, Elisabeth Stelling, Lisa Lasswitz, Antonia P. Gunesch, Martina Kasten, Francisco J. Zapatero-Belinchón, Graham Brogden, Gisa Gerold, Thomas Pietschmann, Virginie Montiel, Jean-Luc Balligand, Federica Facciotti, Emilio Hirsch, Thomas Gausepohl, Husni Elbahesh, Guus F. Rimmelzwaan, Anne Höfer, Mark P. Kühnel, Danny Jonigk, Julian Eigendorf, Uwe Tegtbur, Lena Mink, Michaela Scherr, Thomas Illig, Axel Schambach, Tobias J. Pfeffer, Andres Hilfiker, Axel Haverich, Denise Hilfiker-Kleiner

**Affiliations:** 1 Department of Cardiology and Angiology, Hannover Medical School, Hanover, Germany; 2 Institute of Experimental Virology, TWINCORE, Center for Experimental and Clinical Infection Research Hannover, Hanover, Germany; 3 German Center for Infection Research, Hanover-Braunschweig Site, Braunschweig, Germany; 4 Department of Gastroenterology, Hepatology and Endocrinology, Hannover Medical School, Hanover, Germany; 5 Department of Clinical Microbiology, Virology & Wallenberg Centre for Molecular Medicine (WCMM), Umeå University, Umeå, Sweden; 6 Department of Biochemistry, University of Veterinary Medicine Hannover, Hanover Germany; 7 Pole of Pharmacology and Therapeutics, Institut de Recherche Expérimentale et Clinique, and Cliniques Universitaires Saint-Luc, Université catholique de Louvain (UCLouvain), Brussels, Belgium; 8 Department of Experimental Oncology, European Institute of Oncology IRCCS, Milan, Italy; 9 Department of Molecular Biotechnology and Health Sciences, Molecular Biotechnology Center, University of Torino, Torino, Italy; 10 Research Center for Emerging Infections and Zoonoses (RIZ), University of Veterinary Medicine in Hannover (TiHo), Hannover, Germany; 11 Biomedical Research in Endstage and Obstructive Lung Disease (BREATH), German Center for Lung Research, Hanover, Germany; 12 Institute for Pathology, Hannover Medical School, Hanover, Germany; 13 Institute of Sports Medicine, Hannover Medical School, Hanover, Germany; 14 Department of Hematology, Hemostasis, Oncology and Stem Cell Transplantation, Hannover Medical School, Hanover, Germany; 15 Hannover Unified Biobank (HUB), Hannover Medical School, Hanover, Germany; 16 Institute of Experimental Hematology, Hannover Medical School, Hanover, Germany; 17 Division of Hematology and Oncology, Boston Children’s Hospital, Harvard Medical School, Boston, MA, United States of America; 18 Department of Cardiac, Thoracic, Transplantation and Vascular Surgery, Hannover Medical School, Hanover, Germany; 19 Department of Cardiovascular Complications of Oncologic Therapies, Medical Faculty of the Philipps University Marburg, Marburg, Germany; Instituto Butantan, BRAZIL

## Abstract

The SARS-CoV-2 coronavirus has led to a pandemic with millions of people affected. The present study finds that risk-factors for severe COVID-19 disease courses, i.e. male sex, older age and sedentary life style are associated with higher prostaglandin E2 (PGE2) serum levels in blood samples from unaffected subjects. In COVID-19 patients, PGE2 blood levels are markedly elevated and correlate positively with disease severity. SARS-CoV-2 induces PGE2 generation and secretion in infected lung epithelial cells by upregulating cyclo-oxygenase (COX)-2 and reducing the PG-degrading enzyme 15-hydroxyprostaglandin-dehydrogenase. Also living human precision cut lung slices (PCLS) infected with SARS-CoV-2 display upregulated COX-2. Regular exercise in aged individuals lowers PGE2 serum levels, which leads to increased Paired-Box-Protein-Pax-5 (PAX5) expression, a master regulator of B-cell survival, proliferation and differentiation also towards long lived memory B-cells, in human pre-B-cell lines. Moreover, PGE2 levels in serum of COVID-19 patients lowers the expression of PAX5 in human pre-B-cell lines. The PGE2 inhibitor Taxifolin reduces SARS-CoV-2-induced PGE2 production. In conclusion, SARS-CoV-2, male sex, old age, and sedentary life style increase PGE2 levels, which may reduce the early anti-viral defense as well as the development of immunity promoting severe disease courses and multiple infections. Regular exercise and Taxifolin treatment may reduce these risks and prevent severe disease courses.

## Introduction

The 2019 strain of coronavirus (severe acute respiratory syndrome coronavirus-2 SARS-CoV-2) caused a pandemic with COVID-19 disease affecting millions of people worldwide. Patients with serious disease courses frequently present with severe acute respiratory syndrome that can progress to pneumonia and acute respiratory distress syndrome and shock [1–3]. Systemic inflammation, acute cardiac injury, heart failure, and hypercoagulability are critical complications in COVID-19 disease [1, 4–9]. Identified cell types infected with SARS-CoV-2 include pulmonary epithelial cells, renal cells, cardiomyocytes, endothelial cells and pericytes [10–12].

An increased risk for infection and severe disease courses have been found in association with older age, male sex, cardiovascular comorbidities and air pollution [7, 13–15]. Immunothrombosis integrates innate immunity, activation of platelets, and clotting factors to fight invading pathogens and concurrently promotes inflammation-related tissue damage; in the context of COVID-19 disease, this may explain the systemic hypercoagulability frequently present in COVID-19 patients [8]. Further alterations in the immune system with partially opposing mechanisms have been reported in acute and chronic COVID-19 disease. On one hand, COVID-19 infection appears associated with an upregulation and activation of neutrophils while at the same time lymphocytes are diminished [16]. Reduced lymphocyte populations seem to correlate with more severe organ injury and higher mortality in hospitalized COVID-19 patients [16]. In this regard, T-cell exhaustion [3, 17], reduced circulating and resident B-cell population and loss of germinal centers associated with viral persistence and severe disease courses correlate with high mortality in the acute phase [3, 18, 19]. On the other hand, a growing body of clinical data suggests that a cytokine storm is associated with COVID-19 severity and is also a crucial cause of death from COVID-19 [20–22]. Among potential mechanisms, SARS-CoV-2 induced formation of autoantibodies, tissue and organ injury as well as secondary infection with bacteria and fungi [23, 24].

Prostaglandin (PG) E2, a metabolite of arachidonic acid, is a well-known modulator of viral infection [25]. As such, PGE2 suppresses the adaptive and innate immune systems and promotes infection, e.g., by influenza A virus (IAV) [26, 27]. Moreover, increased circulating PGE2 levels have been associated with reduced immunity in response to IAV vaccination [26, 27]. Interestingly, IAV infection also promotes the production of PGE2 [28]. Cyclooxygenase-2 (COX-2) is a rate-limiting enzyme for the generation of PGE2 and Hydroxyprostaglandin Dehydrogenase 15-(NAD) (HPGD) is an enzyme responsible for the degradation of PGE2 [29]. These findings, supported further by a recent literature review [30] naturally suggested a connection between arachidonic acid metabolism and PGE2 in COVID-19 disease.

We hypothesized that PGE2 modulates the immune response in individuals at risk for severe COVID-19 disease. To test this, we first measured serum PGE2 levels in COVID-19 patients with different levels of disease severity, as well as in subjects with putative risk factors (age, sex, physical fitness) for a severe disease course. To analyze the direct effects of SARS-CoV-2 on PGE2 production, we infected human lung epithelial cells and human precision-cut-lung-slices (PCLS) with SARS-CoV-2. Additionally, we further dissected the mechanisms of PGE2 modulation of immune defense, e.g. through B-cell maturation and the formation of memory cells, and correlated disease severity with lung B-cell content in patient samples. We further tested strategies to reduce PGE2 production or the effect on the above parameters as preventive or therapeutic modalities against severe COVID-19.

## Materials and methods

Unless otherwise stated, chemicals and reagents were all purchased from Sigma-Aldrich.

### Study design

#### COVID-19 study

In this study of 89 patients diagnosed with COVID-19, 41 presented with mild/moderate symptoms and 48 were hospitalized with severe disease. Blood samples were also obtained from male (n = 18) and female subjects (n = 28) (age 18–50 years) from a healthy population established by Hannover Unified Biobank (HUB).

At the time of blood sampling, for 29 patients it was known whether they obtained corticoids or not. Among those n = 14 obtained no corticoids and n = 15 COVID-19 patients with mild and severe disease course received corticoids (Dexamethasone n = 11 or Medrol n = 4). Information on the use of NSAIDs or leukotriene modifiers were not available. None of the healthy controls were under corticoids or nonsteroidal anti-inflammatory drugs (NSAIDs) treatment.

The local ethics committees at Hannover Medical School, Comité d’Ethique Hospitalo-Facultaire of UCLouvain, and the Ethical Committee of IEO has been obtained (IEO1271) approved this study. All patients and healthy control subjects provided written informed consent. The study conforms to the principles outlined in the Declaration of Helsinki.

#### Physical assessment and exercise program in healthy elderly individuals (rebirth 60plus cohort, DRKS00013885)

All subjects in the Rebirth 60plus cohort (DRKS00013885) were initially tested for maximum power output on a cycle ergometer with graded exercise test (GXT). Based on their activities, physical fitness and pathologies, each subject was given an aerobic exercise training program. Once a month, the subjects were contacted by phone to assess training progress and adjust the exercise program, if necessary. All subjects of the Rebirth 60plus study were informed about benefits and risks regarding all study procedures. Height and weight were measured using a scale (seca gmbh & co. kg, Hamburg, Germany). Body fat was measured with a medical Body Composition Analyzer mBCA (seca gmbh & co. kg, Hamburg, Germany). The physical activity was tracked using a GPS watch Forerunner 30 (Garmin Deutschland GmbH, Munich, Germany) and a daily diary where all physical activities were additionally documented. All study procedures were approved by the local ethics committee of Hannover Medical School (Vote #7617) and all subjects provided informed written consent prior to the commencement of the study procedures.

### Blood sampling and blood tests

Blood samples were collected in S-Monovette® tubes containing ethylenediaminetetraacetic acid (EDTA, for plasma) or clot activator (for serum) at the time of hospital admission or at study inclusion (baseline, BL) and at the follow-up (FU) visits after 12 months for the Rebirth 60Plus male and female subjects (age >60 years). Blood samples were also obtained from young male and female subjects (age 18–50 years) from a healthy population established by Hannover Unified Biobank (HUB). Plasma or serum was separated by centrifugation at 1500 rpm for 10 min and aliquots were stored at -80°C. Laboratory workup was performed as part of routine analysis by hospital laboratories for leukocytes, neutrophils, lymphocytes, platelets, CRP and LDH. PGE2 serum and plasma levels were measured using the prostaglandin E2 ELISA kit (abcam ab133021) according to the manufacturer’s protocol.

### Infection of Calu-3 cells with SARS-CoV-2 and Taxifolin treatment

Calu-3 cells (kindly provided by Prof. Pöhlmann, German Primate Center, Göttingen; ATCC Cat# HTB-55; RRID:CVCL_0609) were maintained in Dulbecco’s’ modified Eagle medium and Vero cells (ATCC-CCL-81; Lot 58484194) in Advanced MEM at 37°C and 5% CO_2_. Both media were supplemented with 10% fetal bovine serum, 2 mM glutamine, 0.1 mM non-essential amino acids and 1% Penicillin/Streptomycin. Calu-3 cells (4.5x10^5^ cells/well) were seeded in collagen-coated 24-well plates. For infection, the SARS-CoV-2 (strain SARS-CoV-2/München-1.2/2020/984,p3) [31] kindly provided by Christian Drosten (Charité, Berlin) through the European Virus Archive–Global (EVAg) was used. The isolate was propagated and titrated in Vero cells. Calu-3 cells were pretreated with 100 μM Taxifolin or DMSO (0.15%) for 24 h. Infection with SARS-CoV-2 isolate was performed at a multiplicity of infection (MOI) of 2.0x10^-5^ for 4 h at 37°C in the presence of the compounds. Heat-inactivated virus (15 min, 70°C) served a negative control. After infection, cells were washed twice with PBS before the medium containing the respective compound was added. At 48 h post infection, culture supernatant was collected and heat-inactivated (15 min, 70°C) prior to the detection of PGE2. RNA was isolated from cell lysates using a NucleoSpin RNA kit (Macherey-Nagel) according to the manufacturer’s instructions to analyze virus genome copy numbers, COX-2, HPGD, PTGES2, PTGES3, TNFa and IFNg expression.

### Virus titration in Vero E6 cells for infection of lung slices with SARS-CoV-2

Vero E6 (ATCC CRL-1586) and Vero cells (ATCC CCL-81) were maintained in Eagle’s Minimum Essential Medium (EMEM) (Lonza) supplemented with 25 mM of HEPES (Gibco), 1 × GlutaMAX (Gibco), 100 U/ml penicillin and 100 μg/ml streptomycin. SARS-CoV2 isolate (strain SARS-CoV-2/München-1.2/2020/984,p3) [31] was kindly provided by Christian Drosten. SARS-CoV-2 seed stocks were generated by inoculating Vero E6 (ATCC CRL-1586) at a MOI of 0.001, collecting and aliqouting the culture supernatant at 72 h post infection (hpi), then storing at -80°C in aliquots. SARS-CoV-2 working stocks were generated by an additional passage on Vero cells (ATCC CCL-81) at a MOI of 0.001. Plaque and median tissue culture infectious dose (TCID_50_) assays were performed to titrate the cultured virus after both passages using Vero cells. This stock was used for the *ex vivo* infections of human tissues.

### Infections of precision-cut human lung slices (PCLS) with SARS-CoV-2

PCLS were maintained in DMEM/F12 medium (Gibco, Thermo Fisher Scientific) supplemented with 2 mM of HEPES (Gibco), 1 × GlutaMAX (Gibco), 100 U/ml penicillin and 100 μg/ml streptomycin; this media was also used for virus dilutions and post-infection incubation. On the day of infection, PCLS were rinsed with PBS (without Mg^2+^ and Ca^2+^) then inoculated with 1 × 10^5^ PFU SARS-CoV-2 in 250 μl of media per well in 48-well plates and incubated at 37°C. After 2 h, the inoculum was removed and the PCLS were then cultured in 250 μl of DMEM/F12 medium. At 72 and 120 hpi, supernatants were collected and PCLS were fixed with fixation buffer (4% PFA, 0.1% glutaraldehyde and 200 mM HEPES in ddH_2_O) for 1 h at room temperature followed by 24 h at 4°C.

### QRT-PCR for NSP7 to confirm SARS-CoV-2 infection

SARS-CoV-2 infections in human Calu-3 cells and human lung slices and tissue were verified by NSP7 mRNA expression using qRT-PCR (forward primer: GGG CTC AAT GTG TCC AGT TAC, reverse primer: TTG CCC TGT CCA GCA TT).

### Human lung biopsies from acute COVID-19 patients

Patients with acute COVID-19 (AC, n = 6) have been diagnosed with COVID-19 and were positively tested via PCR as described [4]. All AC patients used in this study showed typical acute respiratory distress syndrome (ARDS) histopathology typical for COVID-19 disease. In addition, NSP7 expression was used to detect SARS-CoV-2 virus in biopsies with the limitation that due to heterogeneous distribution of the virus or already cleared acute infection, PCR is not always positive in every area of the lungs and therefore NSP7 might be not detected.

### Multiplex immunohistochemistry of human lung biopsies

The FFPE sections for each group (Control (Ctrl) n = 3, acute COVID-19 (AC) n = 6, transplant rejected (TR) n = 4) were representatively stained with the manual Opal 7-Color IHC Kit (Akoya Biosciences, Marlborough, MA) as previously described [32]. The primary antibodies CD4 (Cytomed SP35, 1:50), CD8 (Dako M0755, 1:600), CD68 (Dako PGM1, 1:750) and CD20 (Dako M0755, 1:1000) were combined in sequence with the opal fluorophore CD4-Opal520, CD8-Opal570, CD20-Opal540 and CD68-Opal650. The sections were scanned with the Vectra 3 System (Akoya Biosciences, Marlborough, MA). The Regions of Interest (ROIs) were selected representative for small, medium and large vessels for the entire tissue section. The number of analyzed stamps was 43 for Ctrl, 74 for AC and 56 for TR. For the detection of CD20^+^ B cells, the analysis was performed with the inForm Advanced Image Analysis Software Version 2.3.0 (Akoya Biosciences, Marlborough, MA) and ImageJ 1.53c (Wayne Rasband, National Institutes of Health, USA). Statistical analysis was performed using the generalized linear model with Gaussian distribution and weights adjusted according to the number of ROIs per patient.

### Stimulation of human pre-B-cell lines

Human pre-B-cell lines 697 (ACC42 DSMZ collection) and SUP-B15 (ACC389 DSMZ collection) were cultivated in RPMI (Gibco) supplemented with 10% FBS. 5x10^5^ cells per ml were pre-incubated with either the EP1/EP2 receptor antagonist AH6809 (10 μM, Tocris) or the EP4 receptor antagonist GW627368 (10 μM, Tocris) for 2 h. PGE2 (10 μM, Sigma-Aldrich) was added and cells were harvested after 48 h in TRIzol, or stained with trypan blue (Bio-Rad laboratories) and counted for measuring live to dead ratio and cell numbers using the TC20 automated cell counter (Bio-Rad laboratories). Control cells were incubated with dissolvents (DMSO or ethanol (ETHO), 1 μL/ml media). Alternatively, 5x10^5^ per ml 697 and SUP-B15 cells were incubated with 10% human serum from older individuals (>60 y) prior to the commencement of the exercise program at baseline (BL) and after 12M (12M FU) for 48 h and harvested in TRIzol. SUP-B15 cells were incubated with 10% human serum from COVID-19 patients and from healthy controls. Cells were harvested after 48 h in TRIzol.

### PGE2 and prostaglandin D2 (PGD2) detection in supernatants of Calu-3

PGE2 and PGD2 levels in the supernatants of the cell lines Calu-3 (normalized to total RNA content) were measured using the prostaglandin E2 ELISA kit (abcam ab133021) or the prostaglandin D2 ELISA kit (Cayman Chemicals, No. 512031) respectively, according to the manufacturer’s protocols.

### Isolation of RNA and qRT-PCR

Total RNA was isolated with TRIzol (Thermo Fisher Scientific) and cDNA synthesis was performed as described previously [33]. Real-time PCR with the SYBR green dye method (Brilliant SYBR Green Mastermix-Kit, Thermo Fisher Scientific) was performed with the AriaMx Real-Time PCR System (Agilent Technologies) as described [33]. Expression of mRNA levels was normalized using the 2-ΔΔCT method relative to 18S, beta-2-microglobulin (B2M) and glyceraldehyde-3-phosphate dehydrogenase (GAPDH). A list of qRT-PCR primers used in this study is provided in the supplements file S1 Table.

### RNA isolation from formalin fixed and paraffin embedded tissue

RNA isolation from formalin-fixed and paraffin embedded tissue was performed using the Maxwell^®^ RSC RNA FFPE Purification Kit (Promega Corporation, Madison, WI). RNA content was measured by using the Qubit RNA IQ Assay (Thermo Fisher Scientific, Waltham, MA).

### Statistical analyses

Statistical analysis was performed using GraphPad Prism version 5.0a, 7.0 and 8.1.2 for Mac OS X (GraphPad Software, San Diego, CA, USA).

Normal distribution was tested using the D’Agostino normality test or Shapiro-Wilk normality test if the sample was too small for D’Agostino normality test. Continuous data were expressed as mean ± SD or median and interquartile range (IQR), according to the normality of distribution. Comparison between two groups was performed using one sample *t*-test or unpaired two-tailed t-test for Gaussian distributed data and the Mann-Whitney-U test where at least one column was not normally distributed. When comparing more than two groups, ANOVA and Bonferroni’s post hoc test or Dunnett’s post hoc test were used according to the normality of distribution. Categorical variables are presented as frequencies (percentages) and compared using Fisher’s exact test. A two-tailed *P* value of <0.05 was considered statistically significant. Correlation for BMI, BW, body fat content and age was analyzed via ozone correlation analysis by using Pearson correlation coefficients for Gaussian distributions or for nonparametric Spearman correlation coefficients for non-normal distribution.

## Results

### PGE2 levels in healthy individuals in relation to sex and age

**Fig 1:**
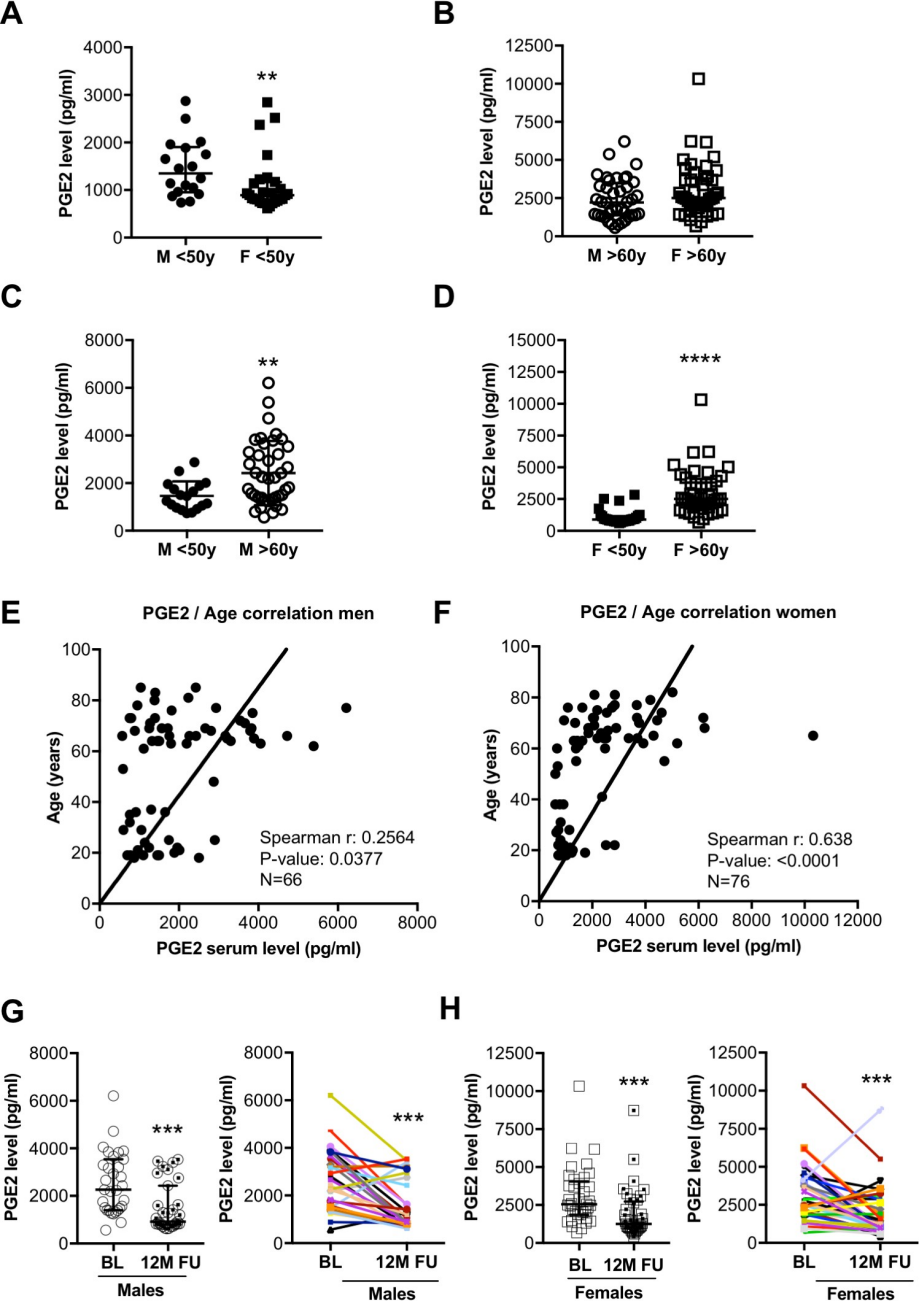
(A) The dot plots summarize circulating serum PGE2 levels (pg/ml) of males (n = 18) and females (n = 28) below the age of 50 years. (B) Dot plots summarize circulating serum PGE2 levels (pg/ml) of males (n = 40) and females (n = 46) over the age of 60 years. (C) The dot plots summarize circulating serum PGE2 levels (pg/ml) of males (n = 18) <50y and males (n = 40) >60y. (D) Dot plots summarize circulating serum PGE2 levels (pg/ml) of females (n = 28) <50y and females (n = 46) >60y. Ozone correlation analysis of serum PGE2 levels with age in (E) males (n = 66, Spearman r: 0.2564, P-value: 0.0377) and (F) females (n = 76, Spearman r: 0.638, P-value: <0.0001). Circulating serum PGE2 levels at baseline (BL) and after 12-months follow-up (FU) following controlled physical training from (G) males (n = 31) and (H) females (n = 37). (A, B, D, G, H) Data are presented as median±IQR, **P<0.01, ***P<0.001, ****P<0.0001, Mann-Whitney-U test. (C) Data are presented as mean±SD, **P<0.01, unpaired two-tailed *t*-test. (E, F) Ozone correlation, Spearman correlation coefficients, two-tailed P value. Underlying data can be found in S1 Data.

In healthy control individuals aged <50, circulating PGE2 levels were higher (P>0.01) in men than in women (Fig 1A). Sex-related differences in circulating PGE2 levels were not observed in older (<60 years) healthy individuals (Fig 1B). Circulating PGE2 levels were markedly higher in older (>60 years) healthy males and females than in respective sex-matched younger (<50 years) individuals (Fig 1C and 1D). Both males and females showed a significant positive correlation of circulating PGE2 levels with age (Fig 1E and 1F), while no correlation with BMI, body weight (BW) or body fat content was observed (S2 Table, S1 Fig). Controlled physical exercise for 12 months reduced PGE2 in elderly male and female individuals compared with their baseline (BL) levels (Fig 1G and 1H and S2 Table). With these indications, we next set to explore whether PGE2 levels changes in COVID-19 and whether differences in PGE2 levels could explain severe disease courses after SARS-CoV-2 infection.

### Circulating levels of PGE2 in COVID-19 patients and age-matched healthy controls

**Fig 2:**
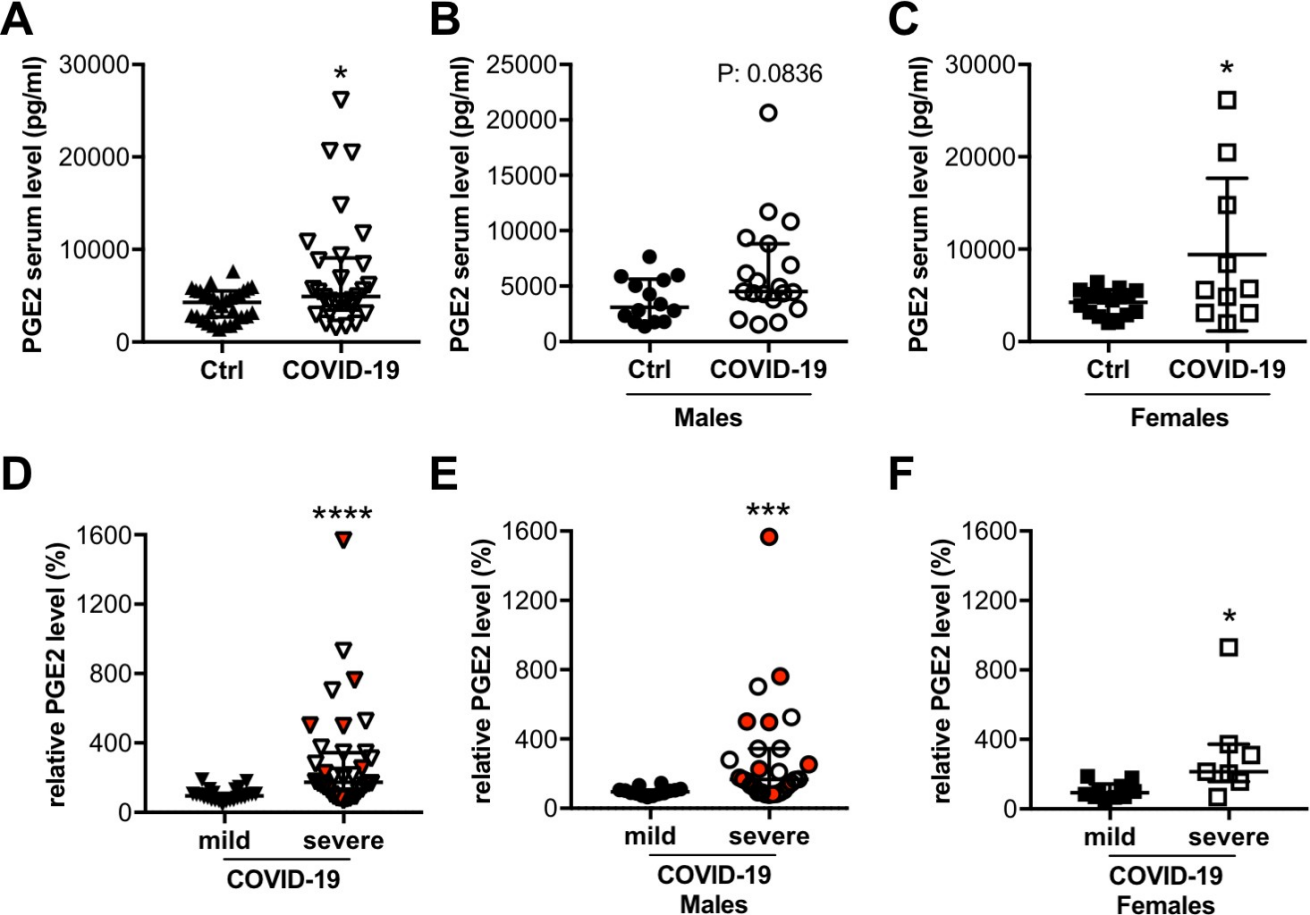
Circulating PGE2 levels are elevated in COVID-19 patients. The dot plots summarize circulating serum PGE2 levels (pg/ml) of (A) COVID-19 patients (n = 29) and healthy controls (n = 31), and separately (B) for males (COVID-19 male patients n = 19; healthy male controls n = 14) and (C) for females (COVID-19 female patients n = 10; healthy female controls n = 17). The dot plots summarize relative circulating plasma PGE2 levels (in %) (D) of patients with severe (n = 36) and mild (n = 24) disease, and separately (E) for males (severe affected males n = 29; mild affected males n = 14) and (F) for females (severe affected females n = 7; mild affected females n = 10); the median of patients with mild disease was set at 100%. Dots representing patients who died with COVID-19 disease are highlighted in red. (A, B, D-F) Data are presented as median±IQR, *P<0.05, ***P<0.001, ****P<0.0001, Mann-Whitney-U test. (C) Data are presented as mean±SD, *P<0.05, unpaired two-tailed *t*-test. Underlying data can be found in S1 Data.

We analyzed PGE2 levels in individuals with mild/moderate (n = 41) and severe (n = 48) COVID-19 disease from hospitals in Hanover (Germany), Milan (Italy) and Brussels (Belgium) and in age-matched healthy controls (n = 31) (Table 1, S3 Table). Clinical data and laboratory characteristics of the COVID-19 patients revealed that the more severely affected patients were significantly older with a higher proportion of males than females compared with the mildly/moderately affected group (Table 1). BMI and diabetes rate are increased in the entire COVID-19 cohort with no significant difference between the mild/moderate and the severe groups (Table 1). In addition, C reactive protein (CRP) was elevated, while the total leukocyte- and neutrophil counts were within the normal range, although some patients displayed markedly increased levels (Table 1). The mean lymphocyte counts (T- and B-cells) were reduced in the majority of COVID-19 patients and were specifically low in patients with severe disease courses (Table 1). Platelets were in the normal range in all COVID-19 patient groups and lactate dehydrogenase (LDH) was increased and highest in the severely affected patients (Table 1). Mortality was 15% for the entire cohort with no patient deaths in the mild/moderate group and 27% of patients dying in the severe disease group who were all of male sex (Table 1). Circulating PGE2 levels were increased in COVID-19 patients at the time of hospital admission compared with healthy controls, and PGE2 levels were significantly higher in the severely affected patients compared with mildly/moderately affected patients (Fig 2A–2F, Table 1). A direct relationship of PGE2 levels to death events was not observed (Fig 2D–2F).

**Table 1:** Summary of clinical data of the COVID-19 patients.

Parameters	COVID-19 patients total	Mild to moderate COVID-19 disease	Severe COVID-19 disease
	(N = 89)	(N = 41)	(N = 48)
Age (years, median ± IQR)	59 (46–68)	51 (40–67)	62 (51–68.75)*
Sex female (%)	30% (27/89)	44% (18/41)	19% (9/48)*
Body weight (kg, median ± IQR)	79.5 (67.75–96.5)	75 (65.5–86.5)	90 (76–100)*
	(n = 42)	(n = 25)	(n = 17)
Body height (cm, mean ± SD)	171.9±9.4	170±9.28	174.6±9.1
	(n = 43)	(n = 25)	(n = 18)
BMI (median ± IQR)	27.2 (23.7–30.2)	26.56 (22.96–28.9)	28 (25.5–31.95)
	(n = 43)	(n = 26)	(n = 17)
Diabetes (%)	28% (11/50)	19% (5/26)	25% (6/24)
Total leucocytes, counts/μl (mean ± SD)	8036±5831	6803±3213	9373±7592
Standard value: 3900–10200 counts/μl	(n = 50)	(n = 26)	(n = 24)
Neutrophils, counts/μl (mean ± SD)	4999±2697	4664±2614	5502±2839
Standard value: 1500–7700 counts/μl	(n = 35)	(n = 21)	(n = 14)
Lymphocytes, counts/μl (mean ± SD)	1114±564	1266±608	**902±428**
Standard value: 1100–4500 counts/μl	(n = 36)	(n = 21)	(n = 15)
CRP mg/L (mean ± SD)	**107±83**	**69.4±55.95**	147.1±89***
Standard value: <5 mg/L	(n = 50)	(n = 26)	(n = 24)
LDH at hospitalization UI/L (median ± IQR)	**363.5 (263.8–518.8)**	**299 (229–375)**	459 (348–659)***
Standard value: <248 UI/L	(n = 48)	(n = 25)	(n = 23)
Platelets at hospitalization 10^3^/μl (mean ± SD)	229±81	235±80	221±83
Standard value: 160–370 10^3^/μl	(n = 50)	(n = 26)	(n = 24)
Mortality (%)	15% (13/89)	0% (0/41)	27% (13/48)***

COVID-19 patients who need hospitalization were defined as severe COVID-19 patients. Body mass index (BMI), C-reactive protein (CRP), lactate dehydrogenase (LDH), leukocytes normal count, neutrophils normal count, and lymphocytes below normal counts, were analyzed at the time of hospital admission in routine clinical lab tests. Standard values of blood parameters were indicated in the parameter column. Values outside the normal range were indicated in bold font. Comparison between the groups of mild and severe COVID-19 was performed using Student’s t-test for Gaussian distributed data (presented as mean ± SD) and the Mann-Whitney-U test where at least one column was not normally distributed (presented as median and interquartile range (IQR)). Categorical variables are presented as frequencies (percentages) and were compared using Fisher’s exact test. *P<0.05, **P<0.01, ***P<0.001 severe COVID-19 vs mild to moderate COVID-19 disease. Underlying data can be found in S1 Data.

### Expression of COX-2 and HPGD and secretion of PGE2 in human lung epithelial cells and precision-cut lung slices infected with SARS-CoV-2

**Fig 3:**
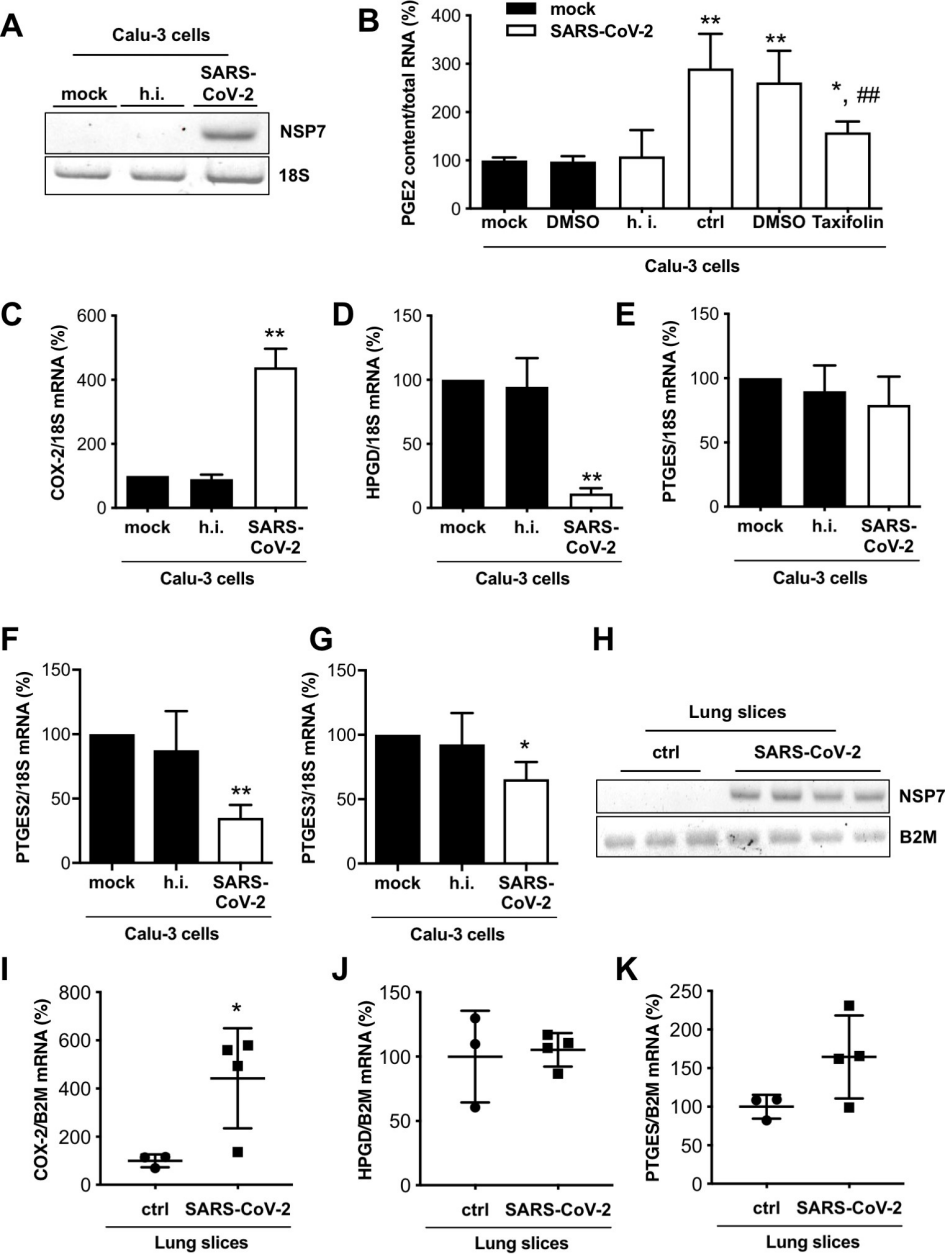
SARS-CoV-2 infection modulates PGE2 secretion and COX-2 and HPGD expression. (A) Representative gel image of NSP7 mRNA expression of Calu-3 cells infected with SARS-CoV-2 and control cells. (B) The bar graph summarizes PGE2 content in supernatants of Calu-3 cells infected with SARS-CoV-2 and treated with Taxifolin (n = 4) compared with untreated mock (n = 6), DMSO control (n = 8) and heat-inactivated (h.i.) SARS-CoV-2 (n = 6) normalized to total RNA. The bar graphs summarize mRNA expressions of (C) COX-2, (D) HPGD, (E) PTGES, (F) PTGES2 and (G) PTGES3 of SARS-CoV-2 infected Calu-3 cells (n = 3). (H) Representative gel image of NSP7 and B2M mRNA expression of SARS-CoV-2 infected lung slices (120 hpi) and control slices. The bar graphs summarize mRNA expressions of (I) COX-2, (J) HPGD and (K) PTGES of SARS-CoV-2 infected lung slices (120 hpi; n = 3 for ctrl, n = 4 for SARS-CoV-2 infection). Data are presented as mean±SD, (B) unpaired two-tailed *t*-test, *P<0.05 vs. mock, **P<0.01 vs. mock, ##P<0.01 vs. SARS-CoV-2 + DMSO. (C-G) One sample t-test, *P<0.05, **P<0.01 vs. ctrl, (I-K) unpaired two-tailed *t*-test, *P<0.05 vs. ctrl. Underlying data can be found in S1 Data and uncropped gel images in S6 Fig.

Next, we investigated whether SARS-CoV-2 would enhance PGE2 production in infected host cells. Human lung epithelial cells (Calu-3 cells) were infected with SARS-CoV-2 (strain SARS-CoV-2/München-1.2/2020/984,p3) [31] and infection was confirmed with qRT-PCR for the SARS-CoV-2 gene encoding nonstructural protein (NSP)7 [34] (Fig 3A). Heat-inactivation of SARS-CoV-2 infected supernatants of Calu-3 cells was not associated with degradation of PGE2 (S2A Fig). Infected cells displayed increased secretion of PGE2, which was specifically prevented by incubation with the PGE2 inhibitor Taxifolin [35, 36] (Fig 3B). The synthesis of other prostaglandins like PGD2 was not altered by Taxifolin in infected Calu-3 cells (S3A Fig). Moreover, Taxifolin treatment was not associated with changes in the proliferation capacity of Calu-3 cells (S3B–S3D Fig).

SARS-CoV-2 infection increased the expression of COX-2 and reduced the expression of the PGE2 degrading enzyme HPGD but did not alter the expression of the PGE synthase (PTGES) in Calu-3 cells (Fig 3C–3E). In contrast, the expression of PGE synthase 2 (PTGES2) and PGE synthase 3 (PTGES3) were significantly reduced by SARS-CoV-2 in Calu-3 lung cells (Fig 3F and 3G). In line with these results, the production of PGD2 was also increased in infected Calu-3 cells (S3E Fig). Additionally, SARS-CoV-2 infection markedly induced the expression of TNFα (644-fold; P<0.05, S2B Fig), which is known to induce COX-2 expression and with this the PGE2 production in human fibroblasts [37]. The expression of IFNγ could not be detected in control or in SARS-CoV-2 infected in human Calu-3 lung cells. Also, the *ex vivo* infection of living human PCLS with SARS-CoV-2 (viral infection analyzed by NSP7 qRT-PCR, Fig 3H) led to an upregulation of COX-2 expression compared with non-infected control slices, while HPGD mRNA levels were unchanged and PGE synthase (PTGES) expression tended to be increased (Fig 3I–3K).

### Effect of PGE2 on the expression of pre-B-cell differentiation and survival factor PAX5 in human pre-B-cells

**Fig 4:**
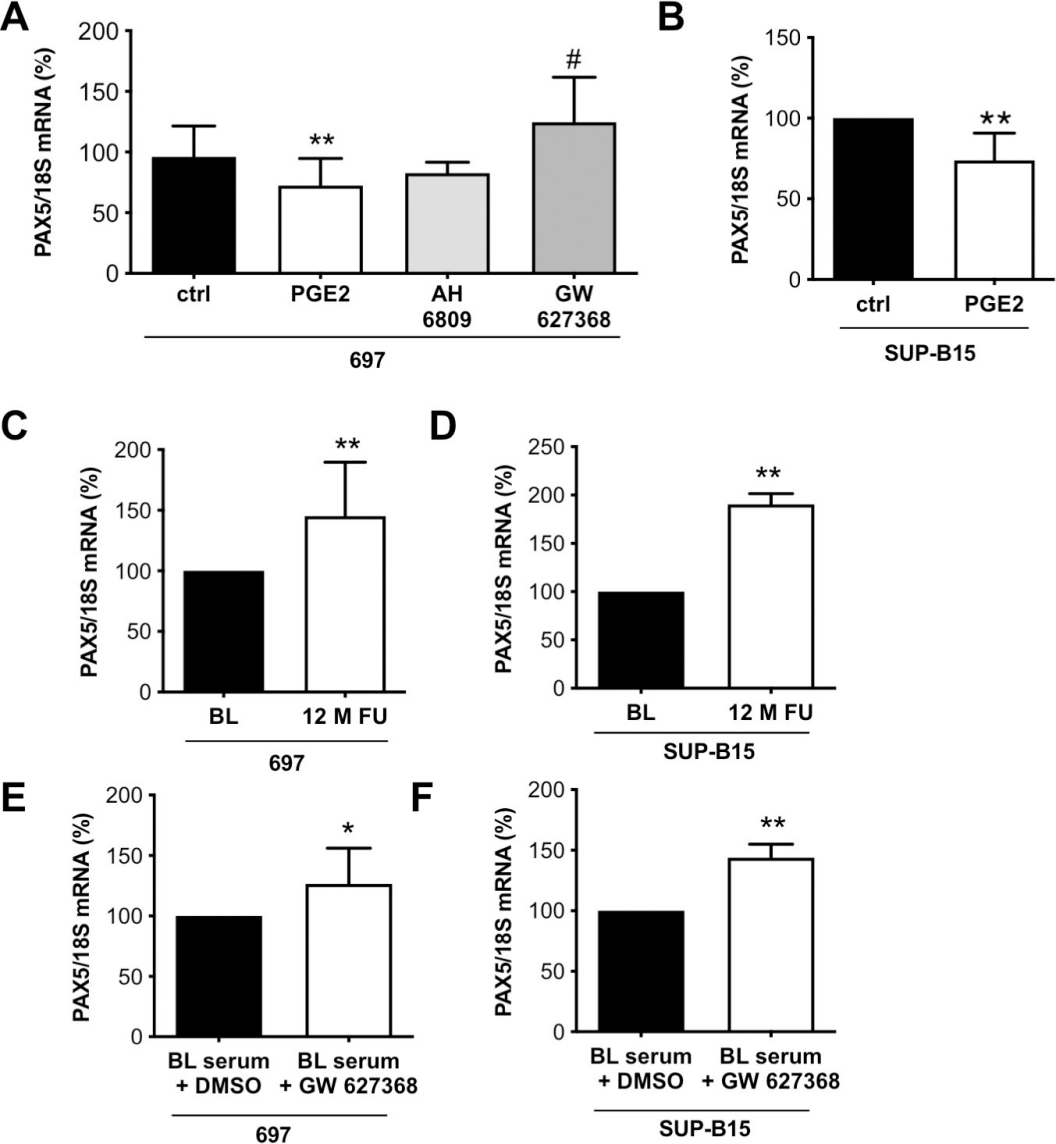
PGE2 stimulation of B-cells modulates the immune response. (A) The bar graph summarizes PAX5 mRNA expression of 697 pre-B-cells treated with AH6809 (10 μM) or GW627368 (10 μM) and PGE2 (10 μM) for 48 h (n = 18 for ctrl and PGE2 treated cells, n = 3 for AH6809 treated cells and n = 6 for GW627368 treated cells). (B) The bar graph summarizes PAX5 mRNA expression of human pre-B-cell line SUP-B15 with PGE2 (10 μM) for 48 h (n = 9). (C) The bar graph summarizes PAX5 mRNA expression of 697 pre-B-cells treated with human serum collected at BL and after 12-months FU of controlled physical training (n = 11). (D) The bar graph summarizes PAX5 mRNA expression of SUP-B15 pre-B-cells treated with human serum collected at BL and after 12-months FU of controlled physical training (n = 4). The bar graph summarizes PAX5 mRNA expression of (E) 697 (n = 11) and (F) SUP-B15 (n = 2) pre-B-cells treated with serum from elderly individuals with high PGE2 levels with and without GW627368 (10 μM). Control pre-B cells were treated with the solvent DMSO. (A) unpaired two-tailed *t*-test, **P<0.01 vs. ctrl, #P<0.05 vs. PGE2, (B-F) One sample t-test, *P<0.05, **P<0.01 vs. ctrl or BL, the mean of ctrl or BL was set at 100%. Underlying data can be found in S1 Data.

PGE2 is known to attenuate the proliferation, differentiation and survival of B-cells [38, 39]. Here, we observed that the addition of PGE2 (10 μM, i.e. 3525 pg/ml), in the range measured in COVID-19 patients’ sera (1300 to >20.000 pg/ml), to two human B-cell precursor lines, 697 and SUP-B15, significantly reduced PAX5 mRNA expression (Fig 4A and 4B). The effect of PGE2 on PAX5 in 697 and SUP-B15 cells could be blocked by co-treatment with the PGE2 receptor 4 (EP4; PTGER4) antagonist, GW627368 but not with the EP2 receptor antagonist, AH6809 (Fig 4A). The expression of PTGER4 in 697 and SUP-B15. Cells was confirmed by qRT-PCR (S4A and S4B Fig). Additionally, PGE2 (10 μM) stimulation was associated with a reduced 697 cell number (51%) compared to control (100%, p<0.01) treated 697 cells. The ratio of live to dead pre-B-cells was not altered through PGE2 stimulation indicating that the decrease in pre-B-cell number is not mediated by enhanced cell death (S4C Fig). However, PGE2 stimulation was associated with a reduced expression of the proliferationmarkers Ki67, TOP2A and TPX2 (S4D–S4F Fig) indicating that it reduces the proliferation capacity of pre-B cells.

### Effect of PGE2 on the expression of inflammatory cytokines TNFα and IFNγ in human pre-B-cells

During SARS-CoV-2 infection upregulation of PANoptosis inducing cytokines, i.e. TNFα and IFNγ have been reported in immune cells [40]. Here, PGE2 stimulation reduced the expression of TNFα in both pre-B cell lines 697 and SUP-B15 (S5A and S5B Fig). The expression of IFNγ was not changed in 697 cells and in SUP-B15 cells, PGE2 reduced its expression (S5C and S5D Fig).

### Effect of serum from elderly individuals before/after physical exercise on PAX5 expression in human pre-B-cells

PAX5 expression was higher in 697 and SUP-B15 pre-B-cells incubated with serum from elderly individuals collected after 12 months of controlled physical exercise compared with their serum before exercise (Fig 4C and 4D and S2 Table). In addition, the EP4 antagonist, GW627368 increased PAX5 in 697 and SUP-B15 pre-B-cells exposed to serum collected before physical exercise, indicating that the suppressive effect is mediated by PGE2-EP4 (Fig 4E and 4F).

### Effect of serum from COVID-19 patients on PAX5 expression in human pre-B-cells

**Fig 5:**
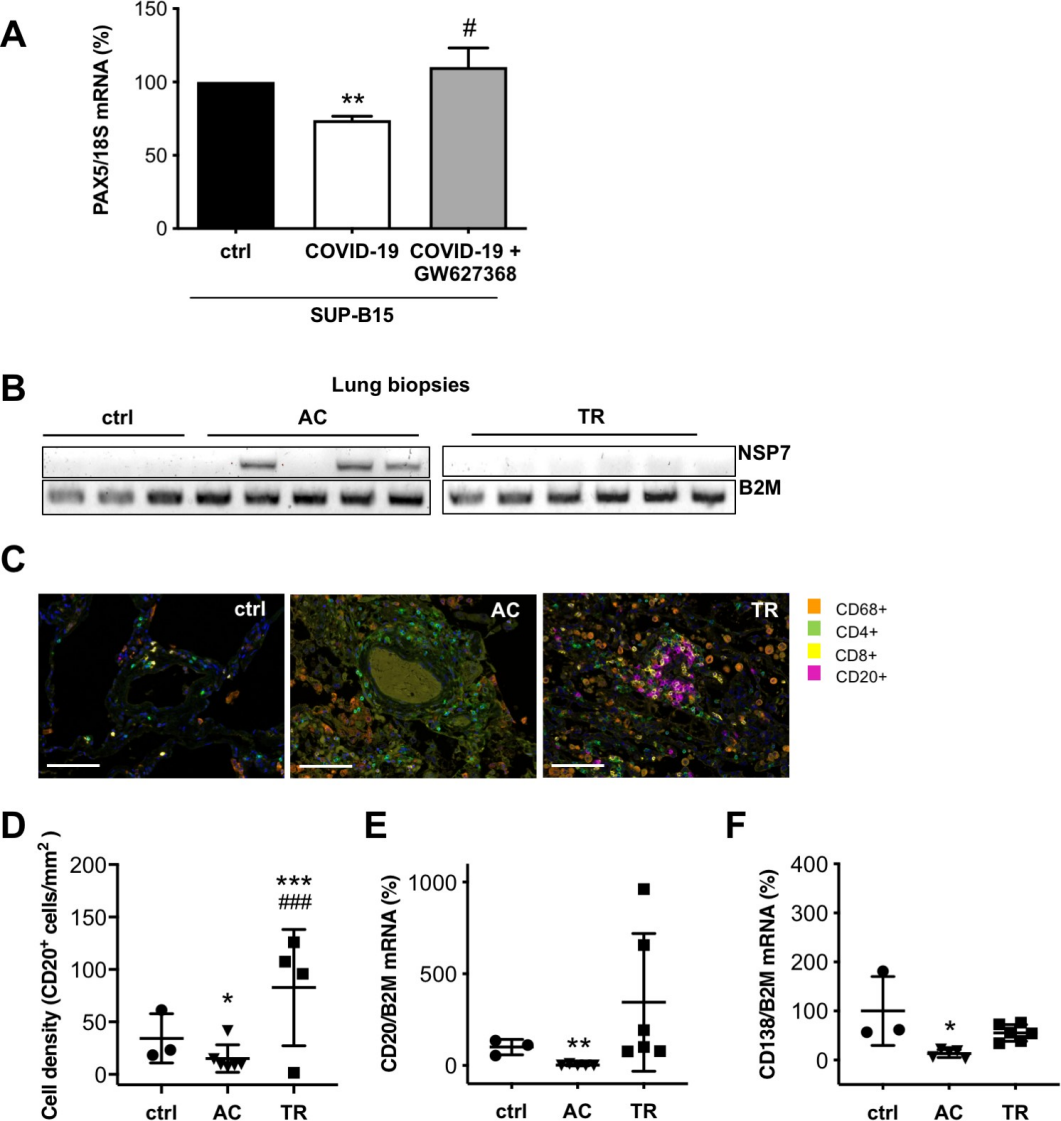
Modulation of the immune response in COVID-19 patients. (A) The bar graph summarizes PAX5 mRNA expression of SUP-B15 pre-B-cells treated with serum from healthy controls (serum pooled from 9 controls) and from COVID-19 patients (serum pooled from 9 COVID-19 patients) incubated with and without GW627368 (10 μM). Control cells were treated with the solvent DMSO (n = 6 wells with control serum and n = 3 wells with serum of COVID-19 patients with and without GW627368). (B) Representative gel image of NSP7 and B2M mRNA expression in control lung tissue (ctrl), in lung tissue of patients with severe acute COVID-19 disease (AC) and in lung tissue obtained after transplant rejection (TR). (C) Immunohistochemical staining for CD68^+^, CD4^+^, CD8^+^ and CD20^+^ immune cells (scale bar: 100 μm), (D) Dot plot summarizing the immunohistological quantification of CD20 positive B-cells per area (mm), dot plots summarize mRNA expression of (E) CD20 and (F) of CD138 in control lung tissue (ctrl), in the lung tissue of patients with severe acute COVID-19 disease (AC) and in lung tissue obtained after transplant rejection (TR). (A) One sample t-test, **P<0.01 vs. ctrl, # P<0.05 vs. serum from COVID-19 patients. (D) Statistical analysis was performed using the generalized linear model with Gaussian distribution and weights adjusted according to the number of ROIs per patient, ***P<0.001 vs. ctrl, ^###^P<0.001 vs AC. (E, F) unpaired two-tailed *t*-test, **P<0.01 vs. ctrl, *P<0.05 vs. ctrl. Underlying data can be found in S1 Data and uncropped gel images in S7 Fig.

Serum from COVID-19 patients with elevated PGE2 levels reduced the expression of PAX5 in SUP-B15 cells compared with serum from healthy controls. Again, this effect was blocked upon co-treatment with the PGE2 receptor 4 (EP4) antagonist, GW627368 (Fig 5A).

### Analyses of B-cells in lungs from patients who died of severe acute COVID-19 disease compared with healthy controls and transplant rejection biopsies

In lung biopsies from patients who died of severe acute COVID-19 disease (AC group, confirmed by qRT-PCR for NSP7, Fig 5B), the signals for CD20 pre-B-cells (qRT-PCR and immunohistochemical quantification) and plasma cells (qRT-PCR for CD138) were barely detectable and lower than in control lung tissue (ctrl) and markedly lower than in lung tissue obtained after transplant rejection (TR, Fig 5C–5F). Lung tissue immunostaining showed increased numbers of CD68^+^ macrophages and CD4^+^ T-cells in AC and TR compared with ctrl lung biopsies (Fig 5C).

## Discussion

The key finding of this study is that PGE2 is elevated in patients with COVID-19 disease, with the highest blood levels observed in those severely affected. Furthermore, SARS-CoV-2 itself upregulates PGE2 in infected host cells and risk factors such as male sex, age and sedentary life style are also associated with higher PGE2 serum levels. Finally, PGE2 impairs the B-cell mediated immune response at least in part by reducing PAX5 while the PGE2 inhibitor Taxifolin attenuates SARS-CoV-2 induced PGE2 production. Moreover, regular exercise also reduces PGE2 levels in elderly subjects, which is associated with increased PAX5 production in B-cells exposed to these sera. Thus, PGE2 may emerge as a modulating factor for disease severity and development of immunity and could therefore be a therapeutic target in COVID-19 prevention and treatment.

**Fig 6:**
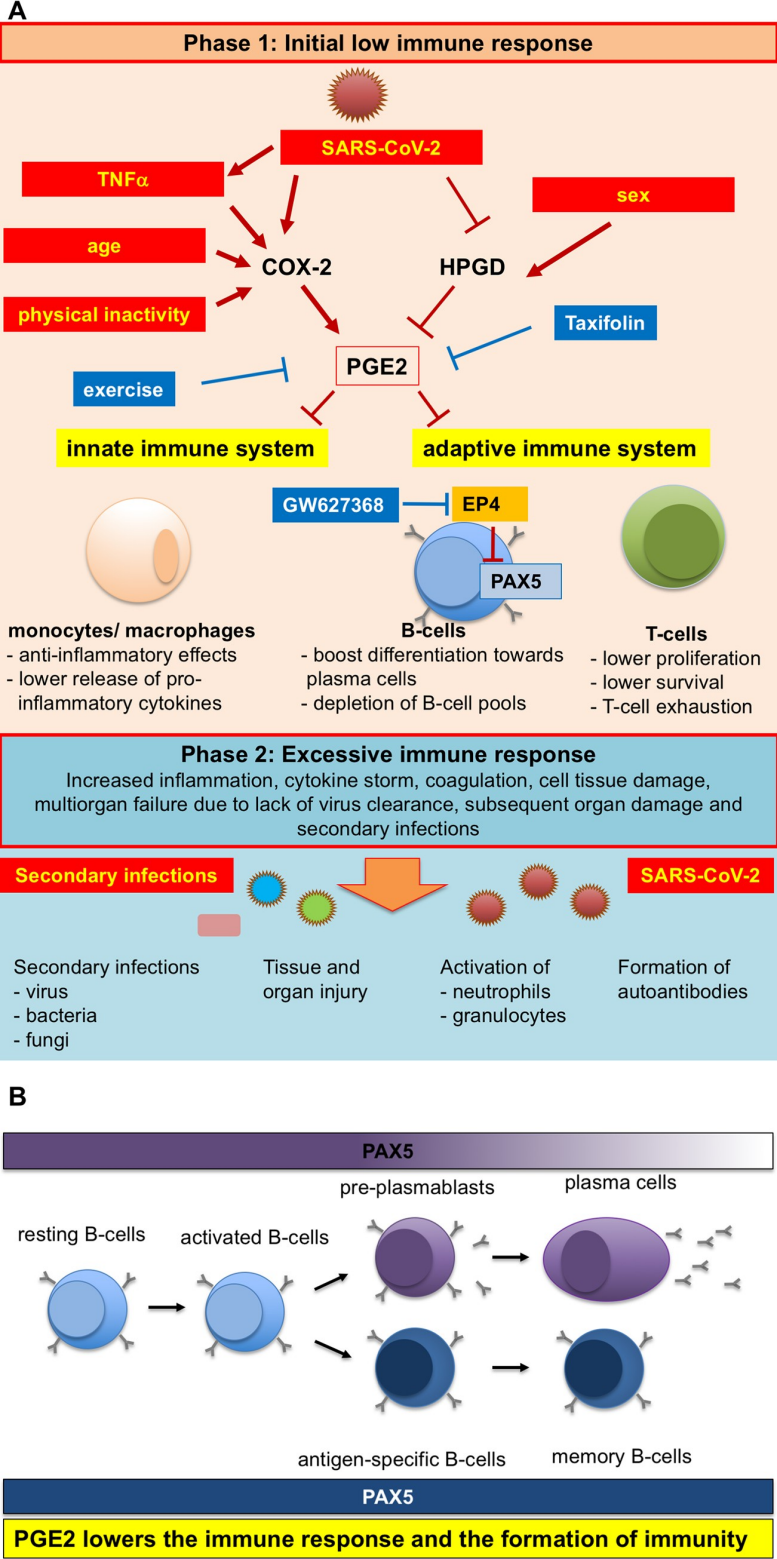
Schematic representation of pleiotropic influences of SARS-CoV-2 infection, physical activity and age on PGE2 levels and the ensuing altered immune response. (A) Modulators of PGE2 synthesis and degradation are SARS-CoV-2 infection, but also physical inactivity, sex and older age, which are all risk factors for more severe COVID-19 disease courses [7, 13–15]. Additionally, SARS-CoV-2 infection induces TNFα expression that is known to mediate increased COX-2 expression [37]. These modulators upregulate the expression of the PGE2-generating enzyme COX-2 and at least in part reduce the expression of the PGE2-degrading enzyme HPGD, which results in increased generation and secretion of PGE2. PGE2 targets the innate immune system (monocytes/macrophages), where it lowers its efficacy to remove pathogens in part by reducing the release of cytokines [46, 59]. Additionally, PGE2 impairs the response of the adaptive immune system against pathogens by lowering proliferation and survival of T-cells and inducing T-cell [3, 52, 60, 61]. Furthermore, PGE2 is impairing the B-cell response to pathogens in part by directly suppressing the B-cell specific transcription factor PAX5 [62]. Increased PGE2 secretion can be prevented by physical exercise and specific PGE2 inhibitors such as Taxifolin. In addition, Taxifolin reduces viral replication. The low immune response (phase 1) may enable the entry of secondary infections with bacteria and fungi and reinfections with SARS-CoV-2 associated with tissue and organ injury, formation of autoantibodies potentially leading to a cytokine storm and an excessive immune response [20–24]. (B) In pre-B-cells, PAX5 is responsible for suppressing other hematopoietic differentiation programs and promotes proliferation, survival and differentiation of pre-B-cells [42–44]. PGE2 reduces PAX5 expression via its EP4 receptor, which not only reduces their survival and proliferation but boosts the differentiation of B-cells towards plasma cells and may even allow transdifferentiation, features that may lead to the cytokine storm but also the depletion of the B-cell pool (and germinal centers) [19, 42]. In addition, since PAX5 is important for the formation memory cells, PGE2 is therefore also lowering the formation of immunity [44, 47]. Blocking the EP4 signaling with the EP4 receptor antagonist GW627368 prevents downregulation of PAX5 in pre-B-cells and may improve viral defense and formation of immunity against SARS-CoV-2.

Since it is known that PGE2 can exert immunosuppressive effects during viral infection [25–27], its elevation might critically reduce the initial defense against SARS-CoV-2 and may thereby lead to more severe disease courses. Interestingly, our data show that the SARS-CoV-2 virus, not only hijacks the host cell gene expression machinery in order to replicate, but also forces infected host cells to produce PGE2 by upregulating the PGE-generating enzyme COX-2, and at least in part by reducing the expression of the PGE2-degrading enzyme HPGD (Fig 6). In line with the upregulation of COX-2 but without a specific upregulation of PGE2 synthases by SARS-CoV-2 in infected human lung cells, the production of another prostaglandin, PGD2, was also increased. However, to study the regulation and role of PGD2 in COVID-19 disease was beyond the scope of the present study and needs further investigation. In addition, we provide evidence that reported risk factors for more severe COVID-19 disease courses, i.e. male sex, age and a sedentary life style [13, 41] are associated with higher PGE2 levels as PGE2 serum levels are higher in men than women, higher in elderly (>60 years) individuals of both sexes than in younger individuals, and PGE2 levels in elderly could be reduced by regular exercise (Fig 6). These findings might explain why males or elderly individuals are more affected than females or younger individuals. Sex-related differences in circulating PGE2 levels appeared to be specific for younger individuals since in the healthy cohort older <60 years no such differences were observed. Whether age-related hormonal changes in older females contributes to the age effect in women needs to elucidated in future studies.

In addition to already known effects of PGE2 on immune cells, we discovered a novel mechanism by which PGE2 in serum from COVID-19 patients specifically impacts on pre-B-cells since PGE2 in the sera of COVID-19 patients reduces the expression of PAX5 in human pre-B-cells via its EP4 receptor. PAX5 is a master regulator of most aspects of the life cycle of B-cells as it represses the transcription of genes required for the development of other hematopoietic lineages and plasma cells and by controlling numerous genes that are required for early development, antigen-receptor recombination, signaling and adhesion [42–44]. Moreover, while high PAX5 expression is necessary for the above described processes, its reduction is important for the final differentiation of short-lived plasma cells and their antibody (AB) production. Thereby, high PGE2 serum levels on one hand reduces the number of pre-B-cells, but on the other hand boosts the terminal differentiation of B-cells towards short-lived plasma cells, two features that on the long run would lead to depleting the B-cell reservoir. This feature may explain why some patients with initially high SARS-CoV-2-directed AB titers but evolving towards a severe disease course display a reduction in germinal centers [19] and reduced B-cell response thereafter. Our findings in postmortem lung tissue of patients who died of COVID-19 are in line with this interpretation. Indeed, we detected reduced CD20^+^ B-cells numbers in COVID-19 lung tissue in comparison with healthy control tissue or with transplant rejection lung biopsies. Likewise, other reports show no significant lymphocyte invasion in cardiac tissue despite the presence of SARS-CoV-2 particles [10, 11]. Additional studies suggest higher risks for severe disease courses in COVID-19 patients with dysfunctional B-cells due to common variable immune deficiencies (CVIDs) [18], while in turn, patients with larger pools of naïve B-cells seem to build a more effective immune response to SARS-CoV-2 [45].

The observed low B-cell signals in lung biopsies from patients who died during acute SARS-CoV-2 infection may also point to loss of these immune cells by PANoptosis (inflammatory cell death). In this regard, Karki et al. reported that during SARS-CoV-2 infection a combination of TNFα and IFNγ could induce PANoptosis [40]. However, we observed the opposite, i.e. PGE2 reduced the expression of TNFα and IFNγ in pre-B-cells, a feature that has also been reported for monocytes and macrophages [46].

Moreover, we found that PGE2 reduces the proliferation of human pre-B-cells, an observation that fits well with the PGE2-mediated reduction of PAX5 and may thereby contribute to rarification of B-cells in infected tissue.

In addition, since we observed that SARS-CoV-2-infected lung cells upregulate TNFα expression and since TNFα is known to induce COX-2 expression, we found one possible mechanism how SARS-CoV-2 may upregulated PGE2 production in infected tissues (Fig 6A) [37].

As reported above, high PGE2 in COVID-19 serum impairs the B-cell mediated immune response at least in part by reducing PAX5. PAX5 expression is also necessary for the development of memory B-cells after follicular B-cells have encountered antigens [44, 47]. In this regard, elevated PGE2 would also reduce the ability of an organism to develop longstanding immunity after COVID-19 infection. Indeed, there are reports on reinfection in individuals with SARS-CoV-2 [48–50] including a recent case report of a patient with a CD20^+^ B-cell acute lymphoblastic leukemia who developed high AB titers against COVID-19 after an initial recovery. However, the patient experienced a viral reactivation after she lost her COVID-19 AB following the administration of rituximab, cytarabine, and dasatinib for her leukemia, and experienced severe COVID‑19 pneumonia with lymphopenia and high inflammatory markers [51]. PGE2 not only affects B-cells, but also promotes T-cell exhaustion and viral expansion through EP2 and EP4, as revealed by recent studies [52] and immunosuppression caused by T-cell depletion and exhaustion have been suggested as contributing to viral persistence and mortality in COVID-19 patients [3].

Based on the suspected crucial role of PGE2 for COVID-19 disease courses, we tested the potential of the PGE2 inhibitor Taxifolin, also known as dihydroquercetin, to limit SARS-CoV-2-induced PGE2 production in human lung cells (Fig 6A). In agreement with our hypothesis that PGE2 contributes to severe COVID-19 disease, Taxifolin significantly reduced PGE2 production in infected lung cells. Additionally, a recent publication on screening for natural inhibitors for SARS-CoV-2 *in silico* identified Taxifolin as a direct inhibitor of the SARS-CoV-2 main protease [53]. Taxifolin is a potent flavonoid with anti-inflammatory activity, which is present as a natural compound in vegetables and fruits and the Siberian larch, *Larix sibirica*, [35, 36]. It is readily available in foodstuffs and could be tested directly in COVID-19 patients. PGE2 synthesis can be inhibited by NSAIDs, which block COX-1 and -2. However, it is known that NSAIDs are interfering with the RAAS [54] and in this context, controversial data have been reported suggesting that NSAIDs may favor SARS-CoV-2 entry by upregulating ACE2 [55, 56]. Moreover, NSAIDs by inhibiting COX-1 and -2 may also reduce the generation of additional prostaglandins, which may have beneficial effects. Therefore, and because the safety of using NSAIDs in the treatment of COVID-19 patients is discussed critical, we decided to use Taxifolin as an alternative treatment strategy. Indeed, we could show that Taxifolin blocked only the SARS-CoV-2-induced PGE2 synthesis but not the synthesis PGD2 in infected lung cells. Inhibition of the microsomal prostaglandin E synthase-1 (mPGES-1) by sonlicromanol (Khondrion; a drug currently in phase 2b studies for mitochondrial disease), may also be beneficial in COVID-19 patients (Fig 6A). Moreover, COVID-19 patients could also benefit from COX-inhibitors such as aspirin and ibuprofen in the early phase of disease as suggested by a recent review [57]. Treatment of mild and severely affected patients with corticoids, like Dexamethasone or Medrol, has been associated with better outcome. Here, we observed that corticoids seem to have no effect on circulating PGE2 levels although number of patients in these subgroup analyses was too low to be conclusive. Finally, we provide evidence that regular physical activity lowers PGE2 in the serum of elderly individuals without COVID-19 infection and may thereby support their immune systems in fighting SARS-CoV-2 infection (Fig 6A).

Thus, known risk factors for severe COVID-19 disease such as age, sex and physical inactivity are associated with elevated PGE2 levels prior infection and may thereby contribute to a reduced immune response at the time of SARS-CoV-2 infection. In addition, the SARS-CoV-2 infection may further compromise the immune response by further upregulating PGE2 in those individuals with pre-existing higher PGE2 levels. Furthermore, it is known that also the exposure to high levels SARS-CoV-2 virus particles contribute to severe COVID-19 disease also in individuals with otherwise low risk factors (for example severe disease cases in nurses and physicians) [58]. As we could demonstrate that SARS-CoV-2-infected host cells produce high levels of PGE2, a massive infection with SARS-CoV-2 virus may lead to high PGE2 secretion and high circulating PGE2 levels, which subsequently reduced the immune response also in individual with otherwise low risk for severe disease.

## Conclusions

In conclusion, our data suggest that PGE2 production, either induced by SARS-CoV-2 infection or determined by endogenous and exogenous risk factors critically influences COVID-19 disease severity, (Fig 6A). Mechanistically, we show that PGE2 specifically targets B-cells by reducing PAX5, a key factor for B-cell proliferation and differentiation (Fig 6A and 6B). Reducing PGE2 levels preventively and/or during COVID-19 disease may therefore provide a valuable therapeutic strategy to prevent and fight SARS-CoV-2 infection and to enhance and prolong immunity.

### Limitations of the study

Limitations of our study include the limited numbers of blood samples from COVID-19 patients and that clinical data on COVID-19 patients, i.e. as C-reactive protein (CRP), lactate dehydrogenase (LDH), leukocytes normal count, neutrophils normal count, and lymphocytes were not available for all patients.

PGE2 synthesis can be blocked by corticosteroids that inhibit the phospholipases or by NSAIDs that inhibit the cyclooxygenase. In this study, at the time of blood sampling a part of the COVID-19 patients with mild or severe disease were treated with corticosteroids or NSAIDs. Information on the use of NSAIDs or leukotriene modifiers were not available. PGE2 levels in those patients might be underestimated, since both medications may reduce PGE2 biosynthesis.

Most individuals in the healthy elderly collective displayed age-related normal BMI and numbers in subgroup with increased or reduced BMI were too low to perform conclusive correlation analyses with PGE2 levels.

Serum and plasma samples have to be stored at -80°C immediately to avoid degradation of PGE2 and to avoid further prostaglandin synthesis by COX-2. For the present study serum and plasma was immediately being processed, frozen and stored at -80°C.

Venipuncture and *ex vivo* platelet activation may alter plasma prostanoid concentrations, a feature that cannot be completely excluded.

## Supporting information

S1 Fig PGE2 serum levels showed no correlation with BMI, BW or body fat content. Ozone correlation analysis of serum PGE2 levels with (A-E) BMI ((A) males: n = 40, Spearman r: -0.1485, P value: 0.3604; (B) males in normal range BMI 25–30: n = 24, Spearman r: -0.1231, P value: 0.5667; (C) males with a BMI >30: n = 9, Spearman r: 0.3167, P value: 0.4101 (D) males with a BMI <25: n = 7, Spearman r: -0.2143, P value: 0.6615 (E) females: n = 45, Pearson r: 0.03956, P value: 0.7964), (F, G) BW (males: n = 40, Spearman r:-0.08246, P value: 0.6130; females: n = 45, Pearson r: 0.05614, P value: 0.7142) and (H, I) body fat content (males: n = 37, Pearson r:-0.03295, P value: 0.8465; females: n = 43, Pearson r: 0.1374, P value: 0.3797) in (A-D, F, H) males and (E, G, I) females. (A-I) Ozone correlation, Spearman or Pearson correlation coefficients, two-tailed P value. Underlying data can be found in S1 Data.
(TIFF)

S2 Fig SARS-CoV-2 infection in Calu-3 cells. (A) Heat-inactivation (h.i.) of PGE2 for 30 min at 70°C compared to untreated PGE2 (ctrl) from the same sample (n = 4). Data are presented as mean±SD, ctrl was set at 100%, one-sample *t*-test. (B) The bar graph summarizes TNFa mRNA expression of SARS-CoV-2 infection in Calu-3 cells in cell culture lysates (n = 3 independent cell culture experiments). Data are presented as mean±SD, mock was set at 100%, *P<0.05 vs mock, ^#^P<0.05 vs h.i., one-way ANOVA, Dunnett post hoc test. Underlying data can be found in S1 Data.
(TIFF)

S3 Fig Taxifolin treatment has no effect on the secretion of PGD2 or the proliferation capacity of Calu-3 cells. The bar graph summarizes PGD2 content in supernatants of Calu-3 cells infected with SARS-CoV-2 and treated for 48 h with Taxifolin (100 μM; n = 12) compared with DMSO control (n = 10). The bar graphs summarize the mRNA expression of the proliferation markers (B) Ki67, (C) TOP2A and (D) TPX2 of Calu-3 cells treated with Taxifolin (100 μM) for 48 h (n = 7 for ctrl and PGE2 treated cells). (E) The bar graph summarizes PGD2 content in supernatants of Calu-3 cells infected with SARS-CoV-2 (n = 6) compared with untreated mock (n = 6) and heat-inactivated (h.i.) SARS-CoV-2 (n = 6) normalized to total RNA. (A-E) Data are presented as mean±SD, (A, B, C) unpaired two-tailed *t*-test, n.s. (D) Mann-Whitney-U test, n.s. (E) mock was set at 100%, **P<0.01 vs mock, ^#^P<0.05 vs h.i., one-way ANOVA, Bonferroni’s post hoc test. Underlying data can be found in S1 Data.
(TIFF)

S4 Fig PGE2 stimulation of pre-B-cells modulates the cell number due to alterations of the proliferation capacity. Representative gel images of PTGER4 and B2M mRNA expression in pre-B-cell lines (A) 697 and (B) SUP-B15. (C) The bar graph summarizes the percentage of live cells of control treated and PGE2 (10 μM) treated 697 cells after 48h stimulation. Total cell number was set at 100%. (D-F) The bar graphs summarize the mRNA expression of the proliferation markers (C) Ki67, (D) TOP2A and (E) TPX2 of pre-B-cells 697 treated with PGE2 (10 μM) for 48 h (n = 5 for ctrl and PGE2 treated cells). (C-E) Data are presented as mean±SD, (C-E) n. s., **P<0.01 vs ctrl, unpaired two-tailed *t*-test. Underlying data can be found in S1 Data and uncropped gel images in S8 Fig.
(TIFF)

S5 Fig PGE2 stimulation of pre-B-cells is not associated with elevated TNFa or IFNg expression. The bar graph summarizes TNFa mRNA expression of (A) 697 or (B) SUP-B18 pre-B-cells treated with PGE2 (10 μM) after 48 h (n = 5). Control cells were treated with the solvent ETHO (n = 5). The bar graph summarizes IFNg mRNA expression of (C) 697 or (D) SUP-B18 pre-B-cells treated with PGE2 (10 μM) after 48 h (n = 5). Control cells were treated with the solvent ETHO (n = 4). (A-D) Data are presented as mean±SD, (A, C, D) n. s., **P<0.01 vs ctrl, unpaired two-tailed *t*-test and (B) **P<0.01, Mann-Whitney-U test. Underlying data can be found in S1 Data.
(TIFF)

S6 Fig The uncropped gel for Fig 3A and 3H. (TIFF)

S7 Fig The uncropped gel for Fig 5B. (TIFF)

S8 Fig The uncropped gel for S4A and S4B Fig. (TIFF)

S1 Data Numerical raw data. All numerical raw data are combined in a single excel file, “S1_Data.xlsx,” this file consists of several spreadsheets and each contains the data of 1 figure or table.
(XLSX)

S1 Table List of human qRT-PCR primers. (DOCX)

S2 Table Summary of clinical data from male and female probands baseline (BL) and after 12 M Follow-Up (FU) controlled exercise (E). Body mass index (BMI) was determined with BMI = bodyweight (BW) / squared height. Body weight, body height, BMI, body fat and activity were analyzed at BL and after 12M FU controlled exercise. Comparison between the groups BL vs 12M FU was performed using Student’s t-test for Gaussian distributed data (presented as mean ± SD) and the Mann-Whitney-U test where at least one column was not normally distributed (presented as median and interquartile range (IQR)). ***P<0.001, ****P<0.00001 BL vs 12M FU. Underlying data can be found in S1 Data.
(DOCX)

S3 Table Summary of clinical data of healthy controls from Fig 1A. (DOCX)
