# Supplementary figures and images for: Impaired immune response mediated by prostaglandin E2 promotes severe COVID-19 disease
Source: PLoS One. 2021 Aug 4;16(8):e0255335. doi: 10.1371/journal.pone.0255335 (PMC8336874; doi:10.1371/journal.pone.0255335)

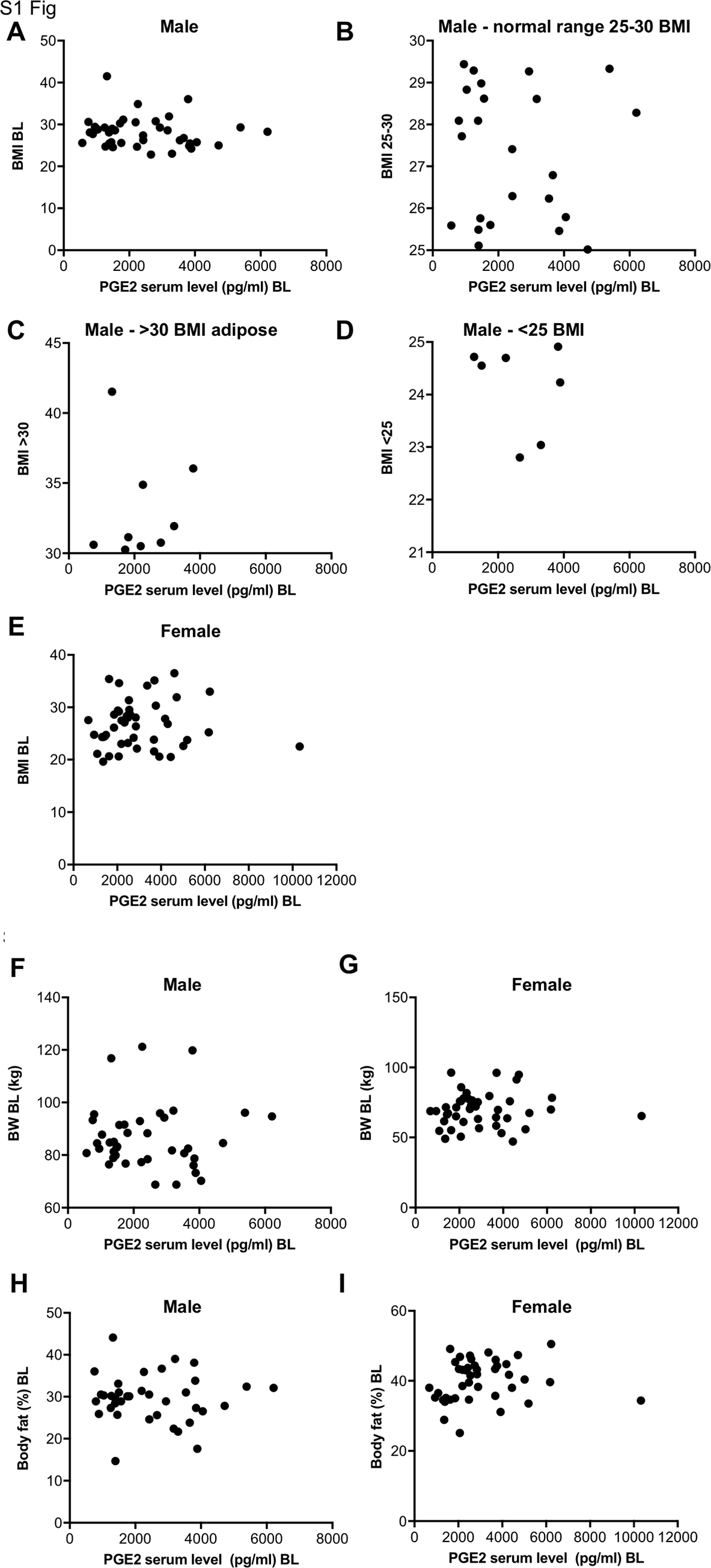

Supplement: S1 Fig — Ozone correlation analysis of serum PGE2 levels with (A-E) BMI ((A) males: n = 40, Spearman r: -0.1485, P value: 0.3604; (B) males in normal range BMI 25–30: n = 24, Spearman r: -0.1231, P value: 0.5667; (C) males with a BMI >30: n = 9, Spearman r: 0.3167, P value: 0.4101 (D) males with a BMI <25: n = 7, Spearman r: -0.2143, P value: 0.6615 (E) females: n = 45, Pearson r: 0.03956, P value: 0.7964), (F, G) BW (males: n = 40, Spearman r:-0.08246, P value: 0.6130; females: n = 45, Pearson r: 0.05614, P value: 0.7142) and (H, I) body fat content (males: n = 37, Pearson r:-0.03295, P value: 0.8465; females: n = 43, Pearson r: 0.1374, P value: 0.3797) in (A-D, F, H) males and (E, G, I) females. (A-I) Ozone correlation, Spearman or Pearson correlation coefficients, two-tailed P value. Underlying data can be found in S1 Data. (TIFF) [file pone.0255335.s001.tiff]

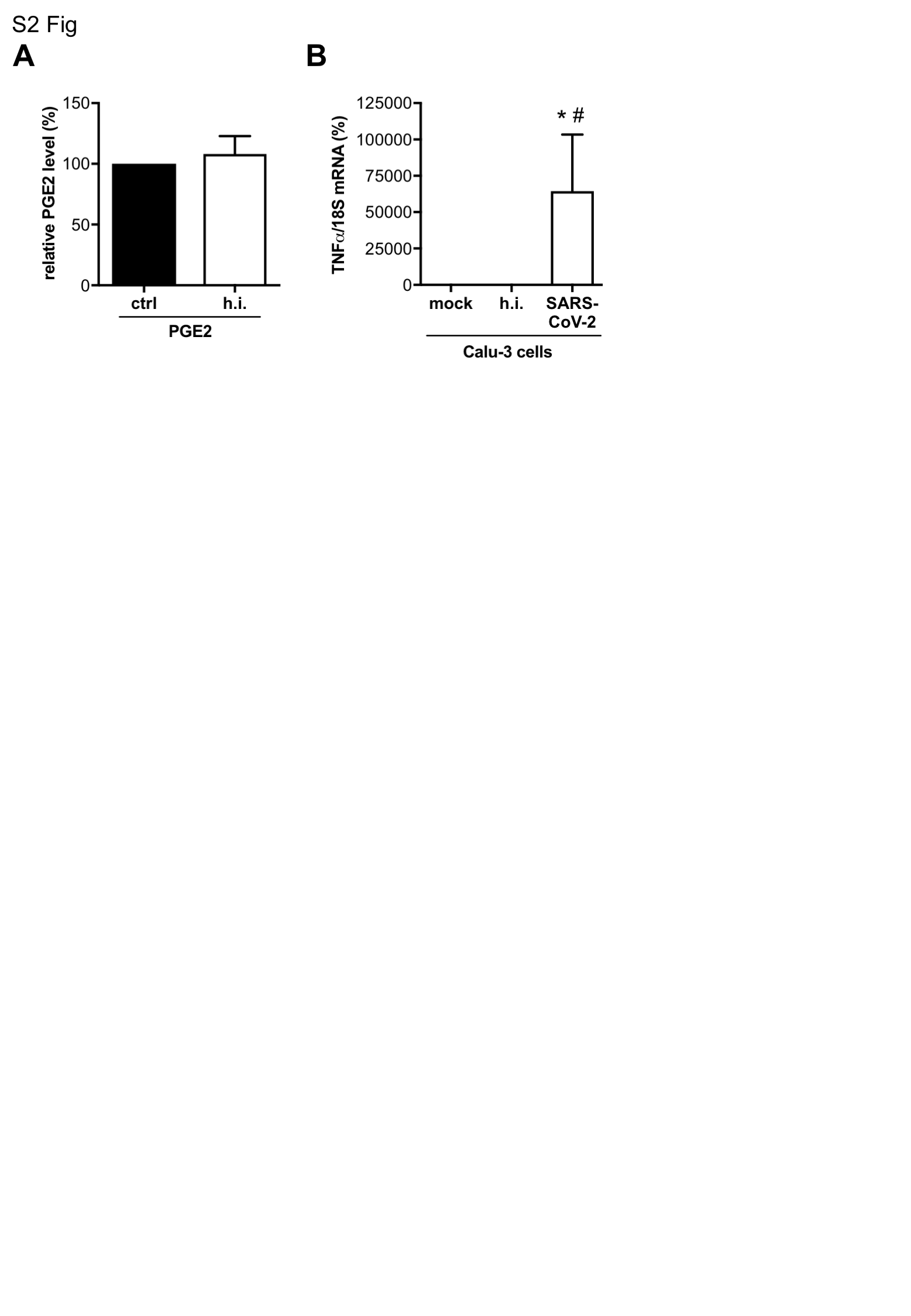

Supplement: S2 Fig — (A) Heat-inactivation (h.i.) of PGE2 for 30 min at 70°C compared to untreated PGE2 (ctrl) from the same sample (n = 4). Data are presented as mean±SD, ctrl was set at 100%, one-sample t-test. (B) The bar graph summarizes TNFa mRNA expression of SARS-CoV-2 infection in Calu-3 cells in cell culture lysates (n = 3 independent cell culture experiments). Data are presented as mean±SD, mock was set at 100%, *P<0.05 vs mock, #P<0.05 vs h.i., one-way ANOVA, Dunnett post hoc test. Underlying data can be found in S1 Data. (TIFF) [file pone.0255335.s002.tiff]

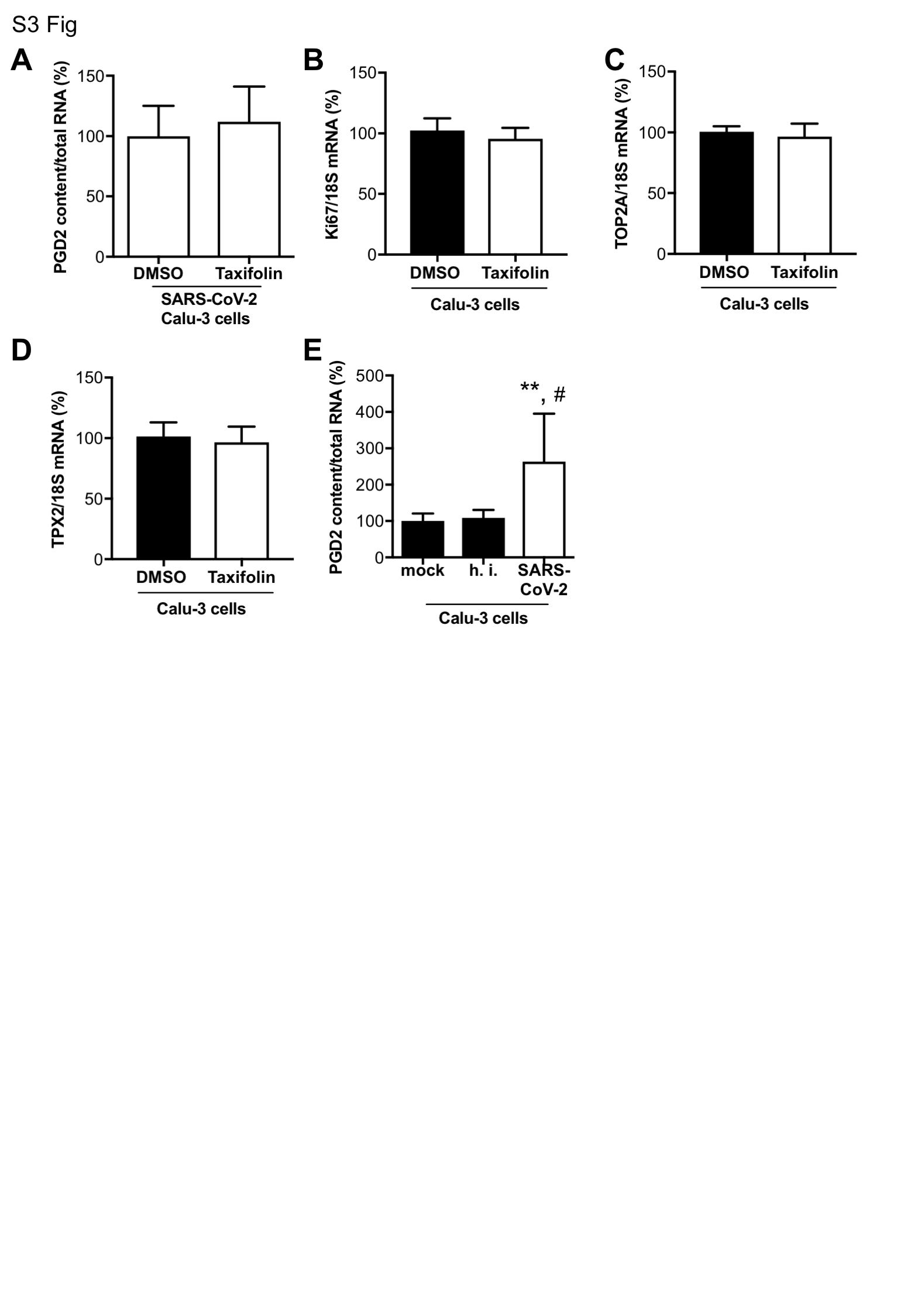

Supplement: S3 Fig — The bar graph summarizes PGD2 content in supernatants of Calu-3 cells infected with SARS-CoV-2 and treated for 48 h with Taxifolin (100 μM; n = 12) compared with DMSO control (n = 10). The bar graphs summarize the mRNA expression of the proliferation markers (B) Ki67, (C) TOP2A and (D) TPX2 of Calu-3 cells treated with Taxifolin (100 μM) for 48 h (n = 7 for ctrl and PGE2 treated cells). (E) The bar graph summarizes PGD2 content in supernatants of Calu-3 cells infected with SARS-CoV-2 (n = 6) compared with untreated mock (n = 6) and heat-inactivated (h.i.) SARS-CoV-2 (n = 6) normalized to total RNA. (A-E) Data are presented as mean±SD, (A, B, C) unpaired two-tailed t-test, n.s. (D) Mann-Whitney-U test, n.s. (E) mock was set at 100%, **P<0.01 vs mock, #P<0.05 vs h.i., one-way ANOVA, Bonferroni’s post hoc test. Underlying data can be found in S1 Data. (TIFF) [file pone.0255335.s003.tiff]

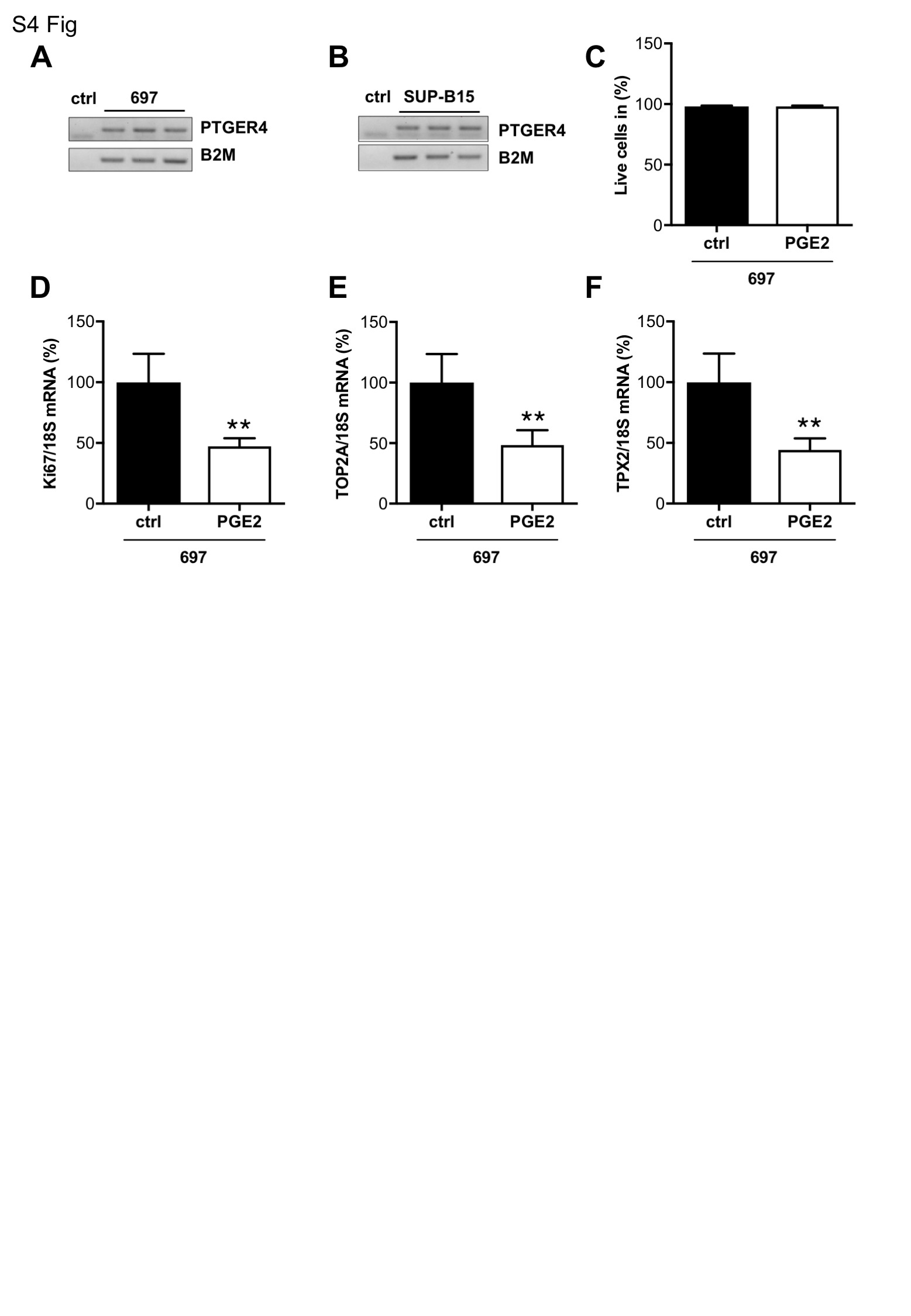

Supplement: S4 Fig — Representative gel images of PTGER4 and B2M mRNA expression in pre-B-cell lines (A) 697 and (B) SUP-B15. (C) The bar graph summarizes the percentage of live cells of control treated and PGE2 (10 μM) treated 697 cells after 48h stimulation. Total cell number was set at 100%. (D-F) The bar graphs summarize the mRNA expression of the proliferation markers (C) Ki67, (D) TOP2A and (E) TPX2 of pre-B-cells 697 treated with PGE2 (10 μM) for 48 h (n = 5 for ctrl and PGE2 treated cells). (C-E) Data are presented as mean±SD, (C-E) n. s., **P<0.01 vs ctrl, unpaired two-tailed t-test. Underlying data can be found in S1 Data and uncropped gel images in S8 Fig. (TIFF) [file pone.0255335.s004.tiff]

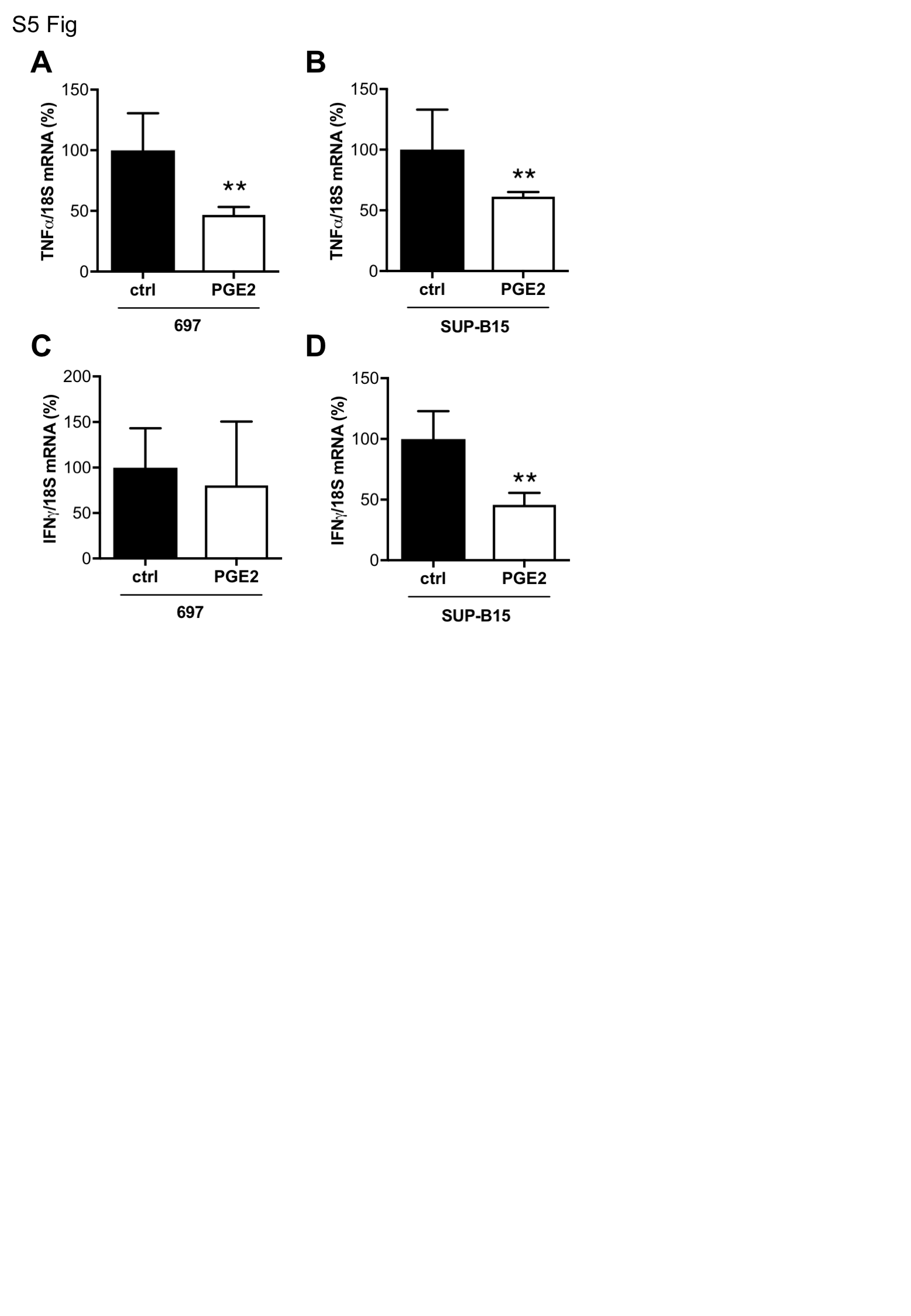

Supplement: S5 Fig — The bar graph summarizes TNFa mRNA expression of (A) 697 or (B) SUP-B18 pre-B-cells treated with PGE2 (10 μM) after 48 h (n = 5). Control cells were treated with the solvent ETHO (n = 5). The bar graph summarizes IFNg mRNA expression of (C) 697 or (D) SUP-B18 pre-B-cells treated with PGE2 (10 μM) after 48 h (n = 5). Control cells were treated with the solvent ETHO (n = 4). (A-D) Data are presented as mean±SD, (A, C, D) n. s., **P<0.01 vs ctrl, unpaired two-tailed t-test and (B) **P<0.01, Mann-Whitney-U test. Underlying data can be found in S1 Data. (TIFF) [file pone.0255335.s005.tiff]

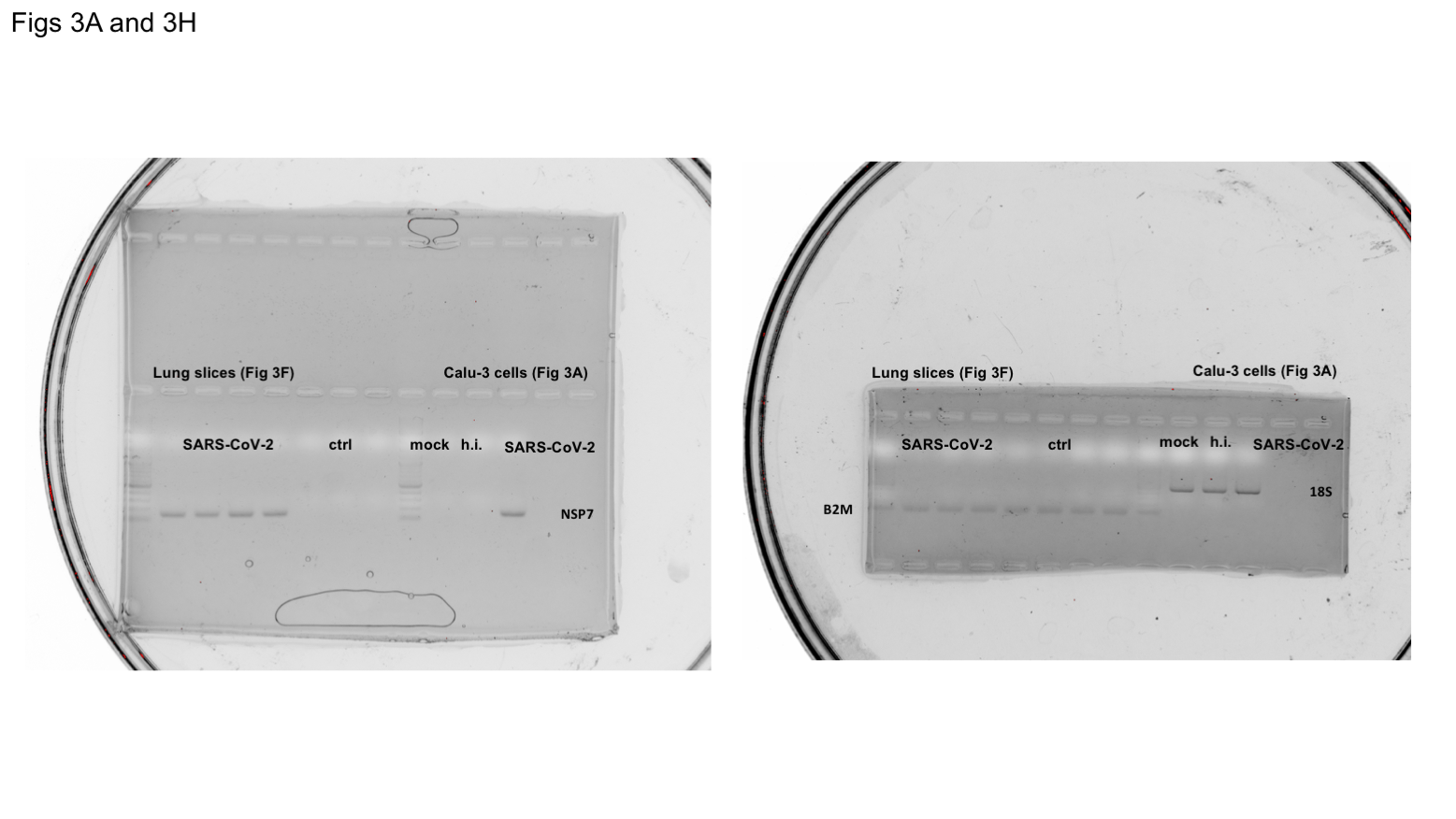

Supplement: S6 Fig — (TIFF) [file pone.0255335.s006.tiff]

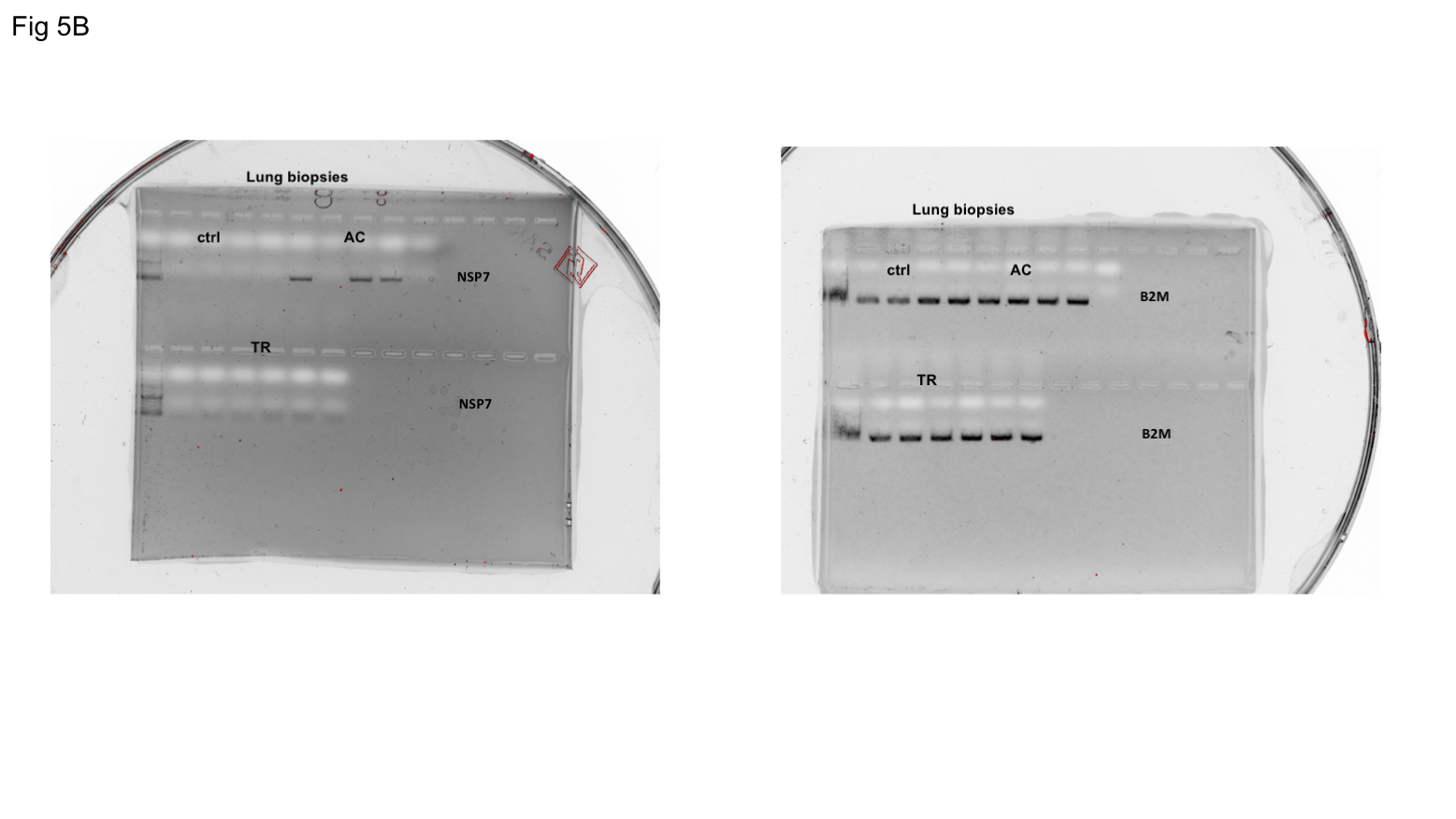

Supplement: S7 Fig — (TIFF) [file pone.0255335.s007.tiff]

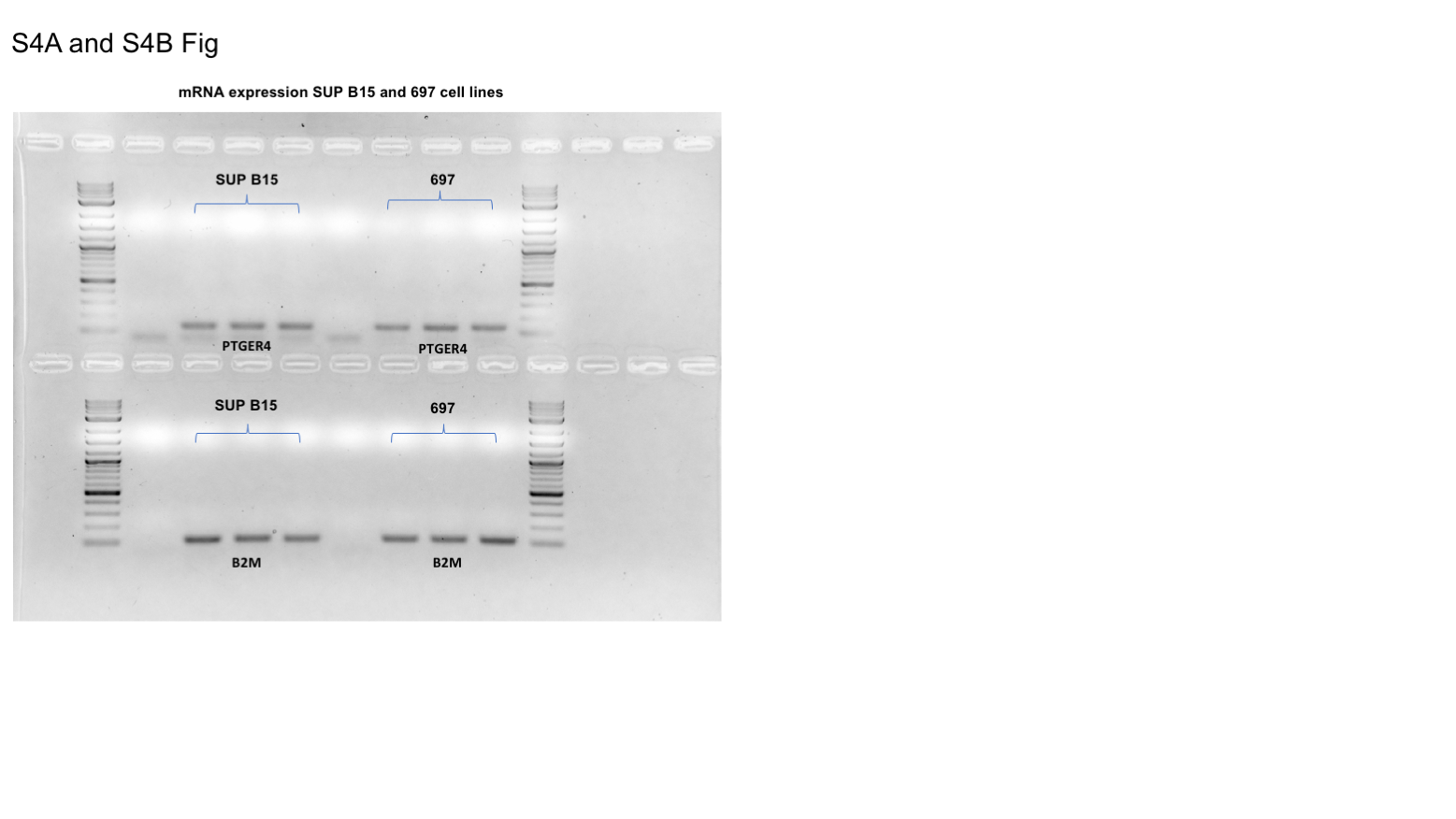

Supplement: S8 Fig — (TIFF) [file pone.0255335.s008.tiff]
