# Supplementary material for: Impaired immune response mediated by prostaglandin E2 promotes severe COVID-19 disease
Source: PLoS One. 2021 Aug 4;16(8):e0255335. doi: 10.1371/journal.pone.0255335 (PMC8336874; doi:10.1371/journal.pone.0255335)
Supplement: S1 Table — (DOCX) [file pone.0255335.s010.docx]

**S1 Table. List of human qRT-PCR primers**

| **Transcript** | **Primer sequence** |
| --- | --- |
| B2M | For: GAGTATGCCTGCCGTGT |
|  | Rev: AATCCAAATGCGGCATCT |
|  |  |
| CD20 | For: AACTGCACCCACTGAACTCC |
|  | Rev: TCTCTTGTTCAGGATCTGAGTCT |
|  |  |
| CD138 | For: GATGGAGGTCCTTCTGCCAC |
|  | Rev: AAAGGTGAAGTCCTGCTCCC |
|  |  |
| COX-2 | For: GAAAACTGCTCAACACCGGAA |
|  | Rev: GCACTGTGTTTGGAGTGGGT |
|  |  |
| HPGD | For: CTTAAGGGCGCCAAGGTAG |
|  | Rev: GCTCATCCAGGGCAGCTTTA |
|  |  |
| IFNg | For: TCGGTAACTGACTTGAATGTCCA |
|  | Rev: TCGCTTCCCTGTTTTAGCTGC |
|  |  |
| Ki67 | For: GAGGTGTGCAGAAAATCCAAA |
|  | Rev: CTGTCCCTATGACTTCTGGTTGT |
|  |  |
| NSP-7 | For: GGGCTCAATGTGTCCAGTTAC |
|  | Rev: TTGCCCTGTTGTCCAGCATT |
|  |  |
| PAX5 | For: TCCGCCAGAGGATAGTGGAA |
|  | Rev: TGGCGACCTTTGGTTTGGAT |
|  |  |
| PTGES | For: CTGCCTCAGGGCCCAC |
|  | Rev: GGAGTAGACGAAGCCCAGGA |
|  |  |
| PTGES2 | For: CTGCTACCCACGCAGAGC |
|  | Rev: GGGACACGTCTTGTACTGGT |
|  |  |
| PTGES3 | For: CACTTTCCTCTTCTCCCCGAC |
|  | Rev: AAGCAGGCTGCATTGTGAAC |
|  |  |
| PTGER4 | For: CTGGTGGTGCTCATCTGCTC |
|  | Rev: AGGATGGGGTTCACAGAAGC |
|  |  |
| TNFa | For: CGCCACCACGCTCTTCTG |
|  | Rev: GCCATTGGCCAGGAGGGC |
|  |  |
| TOP2A | For: CAGTGAAGAAGACAGCAGCAA |
|  | Rev: AGCTGGATCCCTTTTAGTTCCT |
|  |  |
| TPX2 | For: ACATCTGAACTACGAAAGCAT |
|  | Rev: GGCTTAACAATGGTACATCCCTT |
|  |  |
| 18S | For: AGAACGAAAGTCGGAGGTTCG |
|  | Rev: GGACATCTAAGGGCATCACAG |
