# Supplementary material for: Impaired immune response mediated by prostaglandin E2 promotes severe COVID-19 disease
Source: PLoS One. 2021 Aug 4;16(8):e0255335. doi: 10.1371/journal.pone.0255335 (PMC8336874; doi:10.1371/journal.pone.0255335)
Supplement: S2 Table — Body mass index (BMI) was determined with BMI = bodyweight (BW) / squared height. Body weight, body height, BMI, body fat and activity were analyzed at BL and after 12M FU controlled exercise. Comparison between the groups BL vs 12M FU was performed using Student’s t-test for Gaussian distributed data (presented as mean ± SD) and the Mann-Whitney-U test where at least one column was not normally distributed (presented as median and interquartile range (IQR)). ***P<0.001, ****P<0.00001 BL vs 12M FU. Underlying data can be found in S1 Data. (DOCX) [file pone.0255335.s011.docx]

**S2 Table.** **Summary of clinical data from male and female probands baseline (BL) and after 12 M follow-up (FU) controlled exercise (E)**

| **Parameters** | **Male BL**  **(n=31)** | **Male 12 M E**  **(n=31)** | **Female BL**  **(n=39)** | **Female 12 M E**  **(n=39)** |
| --- | --- | --- | --- | --- |
| Age (years, mean ± SD) | 69.6±5.7 | - | 68.6±6.6 | - |
| Body weight (kg, median ± IQR) | 82.5 (77.3-91.4) | 81.2 (77.7-89.9) | 70.3 (64.3-77.3) | 68.7 (62.5-75.9) |
| Body height (cm, mean ± SD) | 175.4±6.1 | 174.4±6.6 | 161.9±6.3 | 162.4±7.1 |
| BMI (median ± IQR) | 26.3 (25.0-29.3) | 26.3 (24.8-29.7) | 26.8 (24.2-29.4) | 25.9 (23.4-28.9) |
| Body fat (%, mean ± SD) | 28.9±6.0  (n=29) | 27.3±6.0  (n=28) | 40.7±5.1  (n=37) | 39.3±5.3  (n=36) |
| Activity (median ± IQR) | 28.1 (17.8-49.7) | 61 (33.4-89.2)*** | 21.1 (15.0-34.0) | 57.5 (30.1-79.0)**** |
