# Supplementary material for: Impaired immune response mediated by prostaglandin E2 promotes severe COVID-19 disease
Source: PLoS One. 2021 Aug 4;16(8):e0255335. doi: 10.1371/journal.pone.0255335 (PMC8336874; doi:10.1371/journal.pone.0255335)
Supplement: S3 Table — (DOCX) [file pone.0255335.s012.docx]

**S3 Table. Summary of clinical data of healthy controls from Fig 2A**

| **Parameters** | **Healthy controls**  **(N=31)** |
| --- | --- |
| Age (years, mean ± SD) | 59.6±14.8 |
| Sex female (%) | 55% (17/31) |
